# A Concerted Vision to Advance the Knowledge of Diabetes Mellitus Related to Immune Checkpoint Inhibitors

**DOI:** 10.3390/ijms24087630

**Published:** 2023-04-21

**Authors:** Maria V. Deligiorgi, Dimitrios T. Trafalis

**Affiliations:** Department of Pharmacology-Clinical Pharmacology Unit, Faculty of Medicine, National and Kapodistrian University of Athens, 11527 Athens, Greece

**Keywords:** autoimmunity, anti-cytotoxic T-lymphocyte antigen 4 (anti-CTLA-4) antibodies, anti-programmed cell death (anti-PD-1) antibodies, anti-programmed cell death ligand 1 (anti-PD-L1) antibodies, diabetic ketoacidosis, immune checkpoint inhibitors, immune-related adverse events, immune-related diabetes mellitus, immunotoxicity, pancreatic islet autoantibodies

## Abstract

The rubric of immune-related (ir) diabetes mellitus (DM) (irDM) encompasses various hyperglycemic disorders related to immune checkpoint inhibitors (ICPis). Beyond sharing similarities with conventional DM, irDM is a distinct, yet important, entity. The present narrative review provides a comprehensive overview of the literature regarding irDM published in major databases from January 2018 until January 2023. Initially considered rare, irDM is increasingly being reported. To advance the knowledge of irDM, the present review suggests a concerted vision comprising two intertwined aspects: a scientific-centered and a patient-centered view. The scientific-centered aspect addresses the pathophysiology of irDM, integrating: (i) ICPi-induced pancreatic islet autoimmunity in genetically predisposed patients; (ii) altered gut microbiome; (iii) involvement of exocrine pancreas; (iv) immune-related acquired generalized lipodystrophy. The patient-centered aspect is both nurtured by and nurturing the four pillars of the scientific-centered aspect: awareness, diagnosis, treatment, and monitoring of irDM. The path forward is a multidisciplinary initiative towards: (i) improved characterization of the epidemiological, clinical, and immunological profile of irDM; (ii) standardization of reporting, management, and surveillance protocols for irDM leveraging global registries; (iii) patient stratification according to personalized risk for irDM; (iv) new treatments for irDM; and (v) uncoupling ICPi efficacy from immunotoxicity.

## 1. Introduction

Reaping the reward of a long and winding journey of investigation, immune checkpoint inhibitors (ICPis)—monoclonal antibodies (mAbs) blocking the immune checkpoints— have signified a turning point in cancer therapeutics [1]. Assigned to ensure immune homeostasis, immune checkpoints are inhibitory immune regulators credited with the maintenance of immune tolerance [1,2,3,4]. Given that the cancer cells hijack the immune checkpoints to enable their escape from immune surveillance, the blockade of the immune checkpoints through ICPis can unleash the immune system to fight cancer [1,2,3,4]. The most exploited immune checkpoints are the cytotoxic T-lymphocyte antigen 4 (CTLA-4), the programmed cell death (PD) 1 (PD-1), and the ligand of the latter (PD-L1) [1,2,3,4]. CTLA-4 is expressed on activated T cells and acts as an inhibitory counterpart of the co-stimulatory molecule CD28 through binding the ligands B7-1 and B7-2 of CD28 with higher affinity than the latter, thereby inhibiting T-cell activation. PD-1 is expressed on multiple immune cells and interacts with its ligands PD-L1 and PD-L2 to mediate coinhibitory signals halting T-cell proliferation, survival, and activation [2,3,4].

The approval of ipilimumab—the archetype anti-CTLA-4 monoclonal antibody (mAb)—by the US Food and Drug Administration (FDA) for treatment of metastatic melanoma in 2011 paved the way for the approval of seven additional ICPis—four anti-PD-1 (nivolumab, pembrolizumab, cemiplimab, dostarlimab) and three anti-PD-L1 (atezolizumab, durvalumab, avelumab,) monoclonal antibodies (mAbs) by the FDA—as of 14 November 2022, to revolutionize the treatment of an increasing list of malignancies [5]. Such a milestone is inevitably related to a constellation of immune-related (ir) adverse events (irAEs), affecting every system [6]. Real-world data leveraging the US FDA Adverse Event Reporting System (FAERS) and the WHO Vigibase (the largest worldwide databases collecting spontaneous reports) designated the endocrine irAEs as the most often reported irAEs until February 2020 [7]. The endocrine irAEs present a unique profile characterized by unpredictable onset, irreversibility, nonspecific symptoms, wide clinical spectrum, and complex diagnostic work-up and management [8,9].

**Figure 1:**
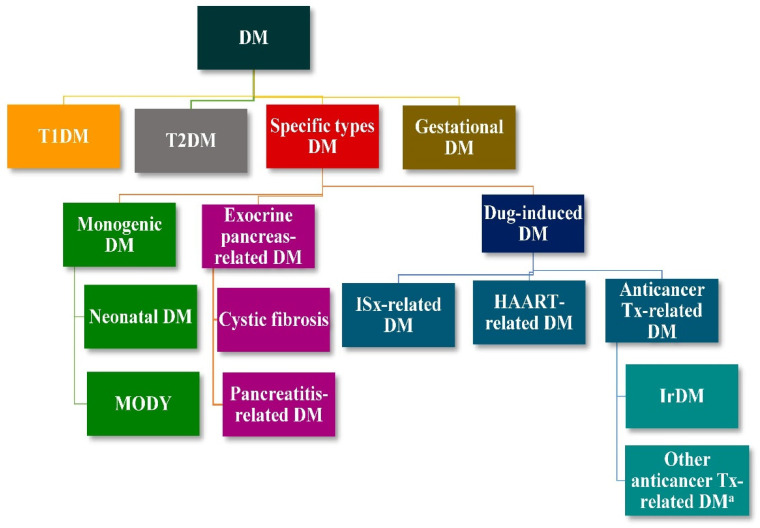
The classification of DM. ^a^ For other anticancer treatments known to cause DM, see Figure 4. Abbreviations: DM, diabetes mellitus; HAART, highly active antiretroviral therapy; irDM, immune-related DM; MODY; maturity onset diabetes of the young; T1DM type 1 diabetes mellitus, T2DM, type 2 diabetes mellitus; Tx, treatment.

Representing an interplay between immuno-oncology and endocrinology, ir diabetes mellitus (irDM) is an intriguing issue [10,11,12]. DM is a metabolic disorder characterized by high plasma glucose levels due to dysfunction of pancreatic beta (β) cells, resulting in impaired insulin secretion, or due to decreased responsiveness of target cells to insulin. DM is classified into four categories, as depicted in Figure 1. Type 1 DM (T1DM) encompasses all autoimmune forms of DM. Type 2 DM (T2DM) is ascribed to insulin resistance and relative lack of insulin [13]. IrDM is included in the category of specific types of DM and in the subcategory of drug-induced DM.

The rubric of irDM encompasses a variety of hyperglycemic disorders related to ICPi. However, to date, the definition of irDM is hampered by varying reporting terms [10,11,12]. Indeed, the clinical spectrum of irDM is wide. New onset of hyperglycemia due to insulin deficiency reminiscent of but not identical to T1DM in patients with no personal history of DM accounts for most cases of irDM. A less common type of irDM is irT2DM [10,11,12]. Exceptionally rare cases of irDM have been recently reported in the setting of two novel irAES: ir pancreatitis [14,15]—termed also as autoimmune pancreatitis (AIP) (Type 3 AIP) [14]—and ir-acquired generalized lipodystrophy (AGL) (irAGL) [16].

IrDM raises several issues that are of paramount importance. First, the pathophysiology of irDM remains unknown, but the almost exclusive association thereof with anti-PD-1/anti-PD-L1 mAbs highlights the role of the disinhibition (inhibition of an inhibitory effect) of the PD-1/PD-L1 interaction. Second, irT1DM presents a distinct autoimmune nature [17]. Third, current data concerning the status of islet autoantibodies and of HLA-related genetic risk are inconclusive. Fourth, irDM has been conventionally considered rare, with an incidence less than 1% [18], but it is currently increasingly reported [19]. Fifth, increased awareness is necessary to avert life-threatening diabetic ketoacidosis (DKA), which is the most common initial presentation of irDM. Finally, close collaboration between endocrinologists and oncologists is essential to promptly diagnose, treat, and monitor irDM.

As the incorporation of ICPi in oncologists’ arsenal evolves at a breathtaking pace [1], the exploitation of this innovative treatment entails the elimination of irAEs, including irDM. To further advance the knowledge of irDM, the present review suggests a concerted vision comprising two interrelated aspects: a scientific-centered and a patient-centered view. The scientific-centered aspect addresses the pathophysiology of irDM. The patient-centered aspect is a personalized clinical practice consisting of four pillars: awareness, diagnosis, treatment, and monitoring of irDM.

## 2. Methods of Data Collection

The key questions in the search query were the following: (1) “Which are the mechanistic underpinnings of the pathophysiology of irDM?” (2) “How common is the irDM?” (3) “How can we predict the irDM?” (4) “How can we effectively manage the irDM?” The literature search was conducted in the following electronic databases: PubMed, Google scholar, Scopus.com, and ClinicalTrials.gov from January 2018 until January 2023. Search terms referred to “diabetes mellitus related to immune checkpoint inhibitors”, “hyperglycemia related to immune checkpoint inhibitors”, “diabetes mellitus in cancer patients”, and “autoimmune diabetes mellitus”. To narrow the search, the authors used the following search blocks: “diabetes mellitus AND cancer”, “diabetes mellitus AND immune checkpoint inhibitors”, “diabetes mellitus AND antibodies against CTLA-4”, “diabetes mellitus AND antibodies against PD-1”, and “diabetes mellitus AND antibodies against PD-L1”. The following types of articles were included in the search: research articles, narrative and systematic reviews, meta-analyses, pharmacovigilance analyses, and guidelines of expert committees. The exclusion criteria for the literature search and study selection were studies published before 2018, editorials, studies in a language other than English, and case reports except for a few exceptional case reports providing unique information. Overall, full articles from the selected literature were retrieved and assessed thoroughly. The aim of the present review is to synthesize the latest data on irDM and suggest a concerted vision that can inform the knowledge of irDM, integrating scientific knowledge with clinical practice.

## 3. The Scientific-Centered Aspect of irDM

The pathophysiology of irDM remains elusive. The prevailing hypothesis for the pathophysiology of irDM concerns the most common type of irDM—irT1DM—and is inspired by the autoimmune nature of conventional T1DM. Recently, the gut microbiome, ir pancreatitis, and irAGL have been implicated in the pathophysiology of irDM, but the mechanistic underpinnings are yet to be clarified.

### 3.1. The Rationale for the Prevailing Hypothesis for the Pathophysiology of irT1DM

The rationale for the prevailing hypothesis for the pathophysiology of irT1DM is an integration of the archetype autoimmune nature of T1DM with the intricacy of immunotoxicity.

Since cancer cells act as “pirates” leveraging immune checkpoints for their own profit, ICPis inevitably cause a disruption to the balance between immunity and autoimmunity, which results in immunotoxicity. The pathophysiology of immunotoxicity is not completely understood, but it reflects an interplay with autoimmunity, genetic susceptibility, and environmental factors. Within this framework, key components of the immune system are implicated in immunotoxicity, namely: (i) T-cell clones responding to self-antigens due to cross-reactivity between tumor antigens or neoantigens and self-antigens in normal tissues, as evidenced by shared T-cell receptor (TCR) sequences between tumors and inflammatory sites involved in irAEs [20,21]; (ii) B cells and autoantibodies [22]; (iii) cells of the innate immune system, as indicated by the association of the neutrophil markers CD177 and CEACAM1 with gastrointestinal irAEs [23]; and (iv) upregulated cytokines and chemokines, as indicated by the integration of 65 cytokines significantly upregulated in patients with severe irAEs into a toxicity score to predict irAEs [20,24,25,26].

Conceived by George Eisenbarth in 1986, the landmark model of the pathophysiology of conventional T1DM outlines the exposure of genetically predisposed individuals to a triggering event that stimulates a process of autoimmune-mediated progressive β-cell destruction, leading to insulin deficiency and, thus, to conventional T1DM. Despite leaving many open questions, this model is still used to inform strategies of precision medicine in conventional T1DM [27].

An integral component of conventional T1DM is a sequence of successive key events, wherein CD4+ and CD8+ T cells are the principal culprits for β-cell destruction. Such key events include: (i) infiltration of pancreatic islets by T cells recognizing various peptides–antigens of β cells; (ii) presentation of peptides–antigens of β cells by antigen-presenting cells (APCs), which interact with self-reactive CD4+ T lymphocytes, which, in turn, activate the self-reactive CD8+T cells; (iii) attack of activated CD8+ T cells against β cells expressing immunogenic self-antigens on major histocompatibility complex class (MHC) I surface molecules, causing β-cell destruction; (iv) exacerbation of β-cell destruction by the innate immune cells (macrophages, natural killer cells, and neutrophils) through release of pro-inflammatory cytokines and reactive oxygen species; (v) promotion of autoimmunity by defects in regulatory T lymphocytes (Tregs), which are CD4+CD25+Foxp3+ T cells that maintain the peripheral tolerance suppressing the autoimmunity via various direct and indirect mechanisms [28,29,30]; and (vi) stimulation of B lymphocytes by activated T cells within the pancreatic lymph node to produce the autoantibodies landmark of T1DM that target β-cell peptides–antigens.

The major self-antigens in conventional T1DM are insulin and its precursor preproinsulin, while the research on other implicated native or post-translationally modified antigens is ongoing [31].

**Figure 2:**
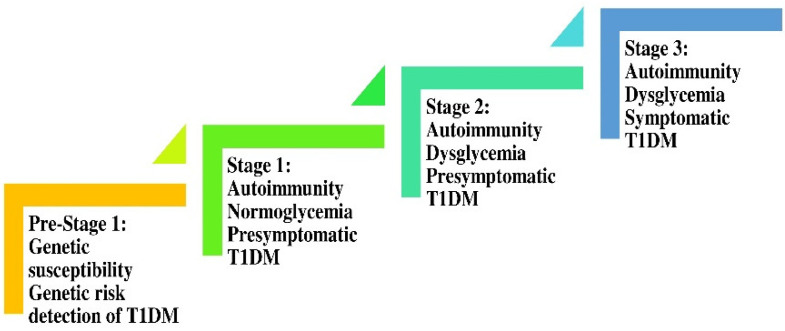
The staging of T1DM according to genetic susceptibility and autoimmunity. Abbreviations: T1DM, type 1 diabetes mellitus.

From a prospective viewpoint, the refinement of the Eisenbarth model leverages the genetic susceptibility and the presence of islet autoimmune antibodies to consider T1DM as a continuum progressing through distinct stages at varying, yet anticipated, rates, as depicted in Figure 2 [32].

Presenting similarities with, but also differences from, conventional T1DM, the prevailing hypothesis regarding the pathophysiology of irT1DM delineates an autoimmune destruction of β cells in genetically predisposed patients, ascribed to an imbalance between immunity and autoimmunity induced by ICPis. As most irDM cases are correlated with anti-PD-1/anti-PD-L1 mAbs, it is postulated that the pathophysiology of irT1DM integrates the role of PD-1 in autoimmunity, the disinhibition of the PD-1/PD-L1 interaction, the genetic susceptibility, and the islet autoantibodies.

#### 3.1.1. The Role of PD-1/PD-L1 Interaction in Autoimmunity

T-cell activation is a finely tuned procedure initiated by the engagement of the TCR by the peptide/major histocompatibility complex (pMHC) ligands, involving positive costimulatory signals, such as the interaction between CD28 on T cells and CD80 (B7.1) and/or CD86 (B7.2) on antigen-presenting cells (APCs). Upon T-cell activation, the induction of coinhibitory checkpoints, among which PD-1 prevails, ensures a balance between immunity and autoimmunity [4]. PD-1 is a type I transmembrane 288-amino acid protein member of the B7/CD28 receptor superfamily. Both ligands of PD-1 (PD-L1 and PD-L2) are type 1 transmembrane proteins with a 40% amino acid identity [2,3,4]. PD-1 is expressed on CD4+ and CD8+ T cells, B cells, monocytes, and subsets of dendritic cells (DCs). Contrary to the broad expression of PD-L1 on hematopoietic and non-hematopoietic parenchymal tissue cells, PD-L2 is expressed only on DCs and subsets of myeloid cells [2,3,4].

The role of PD-1 in the inhibition of T-cell function and proliferation is a complicated issue, the detailed presentation of which is beyond the scope of the present review. Briefly, PD-1 is expressed at a low basal level in naïve T cells, but it is induced early during the process of T-cell activation through the TCR-dictated activation of several transcription factors, including activator protein 1 (AP-1), nuclear factor of activated T cells (NFATs), and nuclear factor-κB (NF-κB), which bind to the cis-regulatory elements of the Pdcd1 gene, initiating the transcription of PD-1. PD-1 can transduce inhibitory signals following the presentation of the peptide–MHC class I complex (pMHCI) ligands by the cells that express PD-1 ligands (PD-L1 and PD-L2), and the subsequent engagement of PD-1 by PD-L1 or PD-L2. PD-1 can transduce signals only after cross-linking with a B-cell receptor or a TCR [4]. Upon PD-1/PD-L1 interaction, PD-1 recruits the phosphatases Src homology region 2 domain-containing phosphatase (SHP)-1 (SHP-1) and SHP-2 to the immune receptor tyrosine-based switch motif (ITSM) in the PD-1 cytoplasmic tail. Such phosphatases can counteract the signaling molecules and/or cascades induced by the interaction of TCR with pMHCI ligands and by the interaction of the costimulatory molecule CD28 with CD80 and/or CD86, such as ZAP70, protein kinase Cθ, phosphoinositide 3-kinase (PI3K)–protein kinase B (AKT) pathway, and RAS-mediated pathways. Overall, the result is decreased activation of critical transcription factors for T-cell activation, proliferation, effector functions, and survival. In addition, PD-1 can inhibit T-cell function via an increase in the expression of the transcription factor basic leucine zipper transcriptional factor ATF-like (BATF), which, in turn, inhibits effector transcriptional programs.

Additionally, PD-1 can induce a distinct time-dependent genetic program affecting glycolysis and oxidative phosphorylation (OXPHOS) through a reduction in the extracellular acidification rate (ECAR) and the basal and stimulated O_2_ consumption rates (OCRs) in CD8+ T cells. Accordingly, the PD-1-induced impairments in the glycolytic and mitochondrial energetics in continuously stimulated CD8+ T cells result in polarization of T cells towards an exhausted state [33]. The exhausted state of CD8+ T cells is integrated through a three-step model comprising persistent antigen presentation, costimulatory inhibition, and chronic inflammation. Exhausted CD8+ T cells gradually lose the effector function, the characteristics of memory T cells, and the self-renewal capacity, while presenting upregulation of inhibitory receptors, metabolic dysregulation, and distinct transcriptional and epigenetic programs [34]. In the context of chronic antigen exposure, PD-1 expression persists and synergizes with key transcriptional factors in the setting of a “chicken–egg” paradigm to induce and reinforce T-cell exhaustion [34,35].

The exhaustion of T cells is the cornerstone of peripheral tolerance, preventing the activation and function of self-reactive T cells, generating a shield to protect islet β cells, among other cells and organs, from autoimmune attack, thereby averting the development of autoimmune T1DM and other autoimmune diseases [36]. The induction and the enhancement of the exhaustion of T cells are currently under evaluation as promising therapeutic tools for T1DM [36,37].

#### 3.1.2. The Role of the Disinhibition of PD-1/PD-L1 Interaction in the Pathophysiology of irT1DM

Considering the established inhibitory role of PD-1/PD-L1 interaction in T-effector cells, the disinhibition of PD-1/PD-L1 interaction can reinvigorate the self-reactive T-effector cells, allowing for autoimmune β-cell destruction, which leads to development of irT1DM. Until more data on the role of the disinhibition of the PD-1/PD-L1 interaction in the pathophysiology of irT1DM are available, we rely on pertinent data concerning the conventional T1DM.

There is ample evidence yielded from non-obese diabetic (NOD) mice indicating that the disinhibition of the interaction between PD-1 expressed on activated T cells and PD-L1 expressed on β cells—but not the disinhibition of the CTLA-4 or of the PD-L2—unleashes the proliferation and the pancreatic infiltration of self-reactive T cells concerning both CD4+ T and CD8+ T cells, thereby causing autoimmune β-cell destruction that results in T1DM [10,11]. These mouse data are beyond the scope of the present review.

Building on mouse data, Colli et al. demonstrated that PD-L1 is expressed on β cells of pancreatic islets of patients with T1DM and is upregulated by the interferon (IFN)-α and IFN-γ via induction of the Janus kinase (JAK)/signal transducer and activator of transcription (STAT)/ Interferon Regulatory Factor 1 (IRF1) pathway—a signaling cascade that integrates the IFN signaling. The authors speculated that there is a dynamic crosstalk between β cells and immune cells during the inflammatory process associated with T1DM, wherein pro- and anti-inflammatory cytokines released by both immune cells and stressed or dying β cells upregulate the PD-L1 expression on human β cells to attenuate the autoimmune attack [38].

Likewise, Osum et al. demonstrated that human β cells upregulate PD-L1 expression to limit the action of self-reactive T cells in response to pancreatic inflammation and the ensuing IFN-γ secretion. The authors assumed that PD-L1 expression intrinsic in β cells can promote the exhaustion of T cells in the pancreas as a self-defensive mechanism [39].

Currently, accumulating clinical data suggest that increased numbers of exhausted self-reactive islet specific CD8+ T cells are a potential predictor of slow progression of T1DM [40].

In a cross-sectional study of patients with T1DM, the proportion of self-reactive islet-specific CD8^+^ T cells expressing an exhausted phenotype discriminated the T1DM patients with slow disease progression who had increased proportion from the T1DM patients with rapid disease progression who had decreased proportion [41].

Wiedeman et al. invented a novel analytical method, DISCOV-R, combining high-content, single-cell mass cytometry with peptide-loaded MHC tetramer staining to characterize the subsets of islet-specific CD8+ T cells. The authors demonstrated that the activated islet-specific CD8+ memory T cells were prevalent in T1DM patients with rapid disease progression, while the exhaustion-like profile of CD8+ memory T cells featuring the expression of multiple inhibitory receptors, including PD-1, limited cytokine production, and reduced proliferative capacity, was prevalent in T1DM patients with slow disease progression [42].

The first evidence of the expression of PD-1 on CD8+ T exhausted memory (Tem) cells in patients with T1DM came from the study of Shan et al. that demonstrated: (i) a significant reduction in frequencies of PD-1+ CD8+ Tem in peripheral blood mononuclear cells (PBMCs) of patients with T1DM (40.73% ± 12.72 versus (vs.) 47.43 ± 15.56, *p* < 0.05); (ii) a significant reduction in PD-1+ CD8+ Tem cells in patients with T1DM with positivity for two or more types of islet autoantibodies compared to patients with positivity for one type of islet autoantibody (13.46% vs. 46.95 ± 12.72%, *p* < 0.05); (iii) a significant reduction in PD-1+ CD8+ central memory T (Tcm) cells in patients with two or more types of autoantibodies compared to other groups [43]. Additional findings from the study of Shan et al. were: (i) positive correlation of the frequencies of PD-1+CD8+ Tem cells with fasting serum C-peptide levels (r = 0.4308, *p* < 0.05) and C-peptide levels 2 h after meal in T1DM patients (r = 0.5723, *p* < 0.01); (ii) negative correlation of the frequencies of PD-1+CD8+ Tem cells with the levels of glycated hemoglobulin (HbA1c) (r = −0.2992, *p* < 0.05); (iii) significant reduction in frequencies of PD-1+CD8+ Tem in the intervention group treated with anti-PD-1 mAbs compared to the control group (14.22 ± 6.455% vs. 27.69 ± 9.837%, *p* < 0.05) [43].

Taken together, the expression of PD-1 on CD8+ Tem protects the host against islet autoimmunity, while the disinhibition of PD-1/PD-L1 interaction through anti-PD-1/anti-PD-L1 mAbs may decrease the exhaustion of T cells, thereby unleashing islet autoimmunity.

Another potential mechanism through which the disinhibition of PD-1/PD-L1 interaction may allow for the reactivation of self-reactive islet-specific T cells is the counter-regulation of the immunosuppressive effect of Tregs. This hypothesis is sustained by the identification and functional characterization of CD8+ Treg cells in T1DM patients, revealing a new CD8+ Treg cell population, which is defective—i.e., less immunosuppressive—due to lower expression of PD-1, pointing to the immunosuppressive role of PD-1 in Tregs [44].

**Figure 3:**
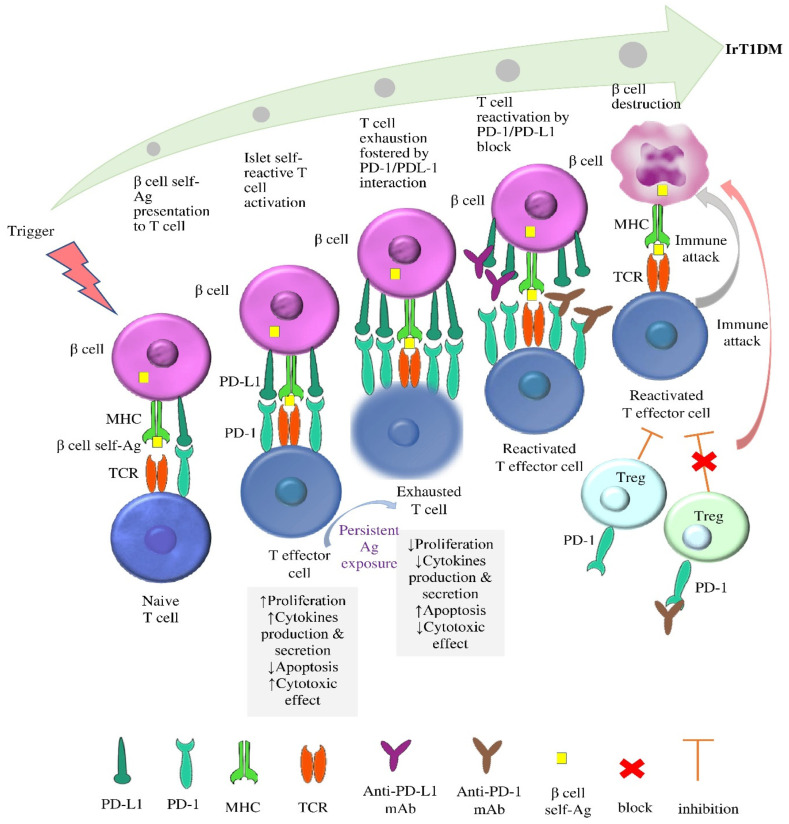
The postulated role of the disinhibition of PD-1/PDL-1 interaction in the pathophysiology of irT1DM.An unknown trigger, presumably environmental, can lead to β-cell stress, stimulating the induction of an immune attack against β cells through PD-L1 expression on β cells along with the recruitment of T cells expressing PD-1. PD-1 expression remains at a low basal level in naïve T cells, but it is induced early during the process of T-cell activation after the engagement of the TCR by the self-antigen/major histocompatibility complex ligand. Interaction of PD-L1 expressed on β cells with PD-1 expressed on activated T-effector cells is known to exert an inhibitory role, enhancing self-tolerance. As the chronic antigen exposure persists, PD-1 expression also persists and PD-1/PDL-1 interaction fosters the transition of T-effector cells to T-cell exhaustion, which is featured by the progressive loss of T-cell functions, averting islet autoimmunity. The disinhibition of PD-1/ PD-L1 interaction by anti-PD-1 or anti-PD-L1 mAbs reinvigorates T cells and allows them to attack β cells, resulting in β-cell destruction and eventually in T1DM. PD-1 is also expressed in T-regulatory cells, which are known to exert an immunosuppressive role inhibiting the function of reactivated T-effectors cells. Thus, inhibition of T-regulatory cells through anti-PD-1 mAbs can enhance the function of reactivated T-effector cells, allowing them to attack β cells, resulting in β-cell destruction and eventually in TIDM. Abbreviations: Ag, antigen; anti-PD-1 mAbs, monoclonal antibodies against programmed cell death (PD) 1 (PD-1), anti-PD-L1 mAbs, antibodies against the ligand of PD-1 (anti-PD-L1), MHC, major histocompatibility complex; PD-1, programmed cell death PD-L1, ligand of PD-1; TCR, T-cell receptor; Treg, T-regulatory cell.

Overall, the available data concerning the role of PD-1/PD-L1 in conventional DM suggest a unifying hypothesis for the pathophysiology of irT1DM comprising two key events. The first event is the exposure of genetically predisposed individuals to unknown, presumably environmental, triggers, which cause β-cell stress and stimulate the priming of an immune response through PD-L1 expression on β cells along with recruitment of CD8+ T cells expressing PD-1, which, in turn, interacts with the PD-L1 expressed on β cells to enhance self-tolerance and attenuate islet autoimmunity. As the chronic antigen exposure persists, PD-1 expression also persists and PD-1/PD-L1 interaction fosters the CD8+ T cell exhaustion that limits islet autoimmunity. The second event is the disinhibition of PD-1/ PD-L1 interaction, which renders β cells victims of reinvigorated CD8+ T cells, resulting in β-cell destruction and eventually T1DM [10,45]. Anti-PD-1 mAbs may also enhance the reinvigoration of CD8+ T cells through disinhibition of the immunosuppressive role of PD-1 in Tregs [44]. The postulated role of the disinhibition of PD-1/PD-L1 interaction in the pathophysiology of irT1DM is illustrated in Figure 3.

Immunohistochemical evidence in two patients with irDM is supportive of the hypothetical pathophysiology of irDM. Yoneda et al. conducted immunohistochemical analysis of the non-tumoral pancreas of a cancer patient with a history of pre-existing T2DM who developed irT1DM related to a combination of anti-CTLA-4 with anti–PD-1 mAbs administrated for renal carcinoma pancreatic metastasis. The analysis showed: (i) profound T-lymphocyte infiltration around islets and exocrine region with predominance of CD8+ T lymphocytes; (ii) limited residual β cells in the pancreas; and (iii) negativity of PD-L1 expression on β cells in most but not all pancreatic islets. The authors considered these findings reflective of the β-cell injury in the setting of irT1DM [46]. The absence of macrophages in the study of Yoneda et al. should be interpreted cautiously in view of the personal history of pre-existing T2DM and the pancreatic metastasis of the patient [47].

Mazzucato et al. provided autoptic evidence from the examination of the pancreas of a 64-year-old patient who developed irDM 10 weeks after the initiation of pembrolizumab for metastatic non-small-cell lung carcinoma. Expression of PD-L1 was demonstrated in 42% of specific endocrine tissue. The pancreatic histology showed a pattern of typical insulitis related to ICPis similar to the insulitis in conventional T1DM [48].

#### 3.1.3. Genetic Susceptibility to irDM

Genetic susceptibility to irDM shows similarities with, but also differences from, that to conventional autoimmune T1DM.

The genetic susceptibility to conventional T1DM has long been pursued. The human leukocyte antigen (HLA) system—a complex of genes on chromosome 6 in humans encoding cell-surface proteins credited with the regulation of the immune system—is responsible for 30–50% of the genetic risk of conventional T1DM. The HLA class II haplotypes DRB1*0301–DQB1*0201 (DR3–DQ2) and DRB1*0401–DQB1*0302 (DR4–DQ8) are correlated with approximately 50% of conventional T1DM heritability. The HLA genotype associated with the highest risk for T1DM is the heterozygous DR3/4 genotype. The HLA class II DRB1*1501 and DQA1*0102–DQB1*0602 haplotypes are considered protective against conventional T1DM.

Beyond the HLA class II genes, several HLA class I genes and non-HLA genes implicated in immunity or β-cell function have been linked to genetic susceptibility to conventional T1DM [49]. So far, approximately 60 single nucleotide polymorphisms (SNPs) in multiple non-HLA genes have been reported to confer genetic susceptibility to conventional T1DM [17,50,51].

In a review of irDM over a 6-year period at two academic institutions, predominance of HLA-DR4 was reported in 76% of patients with irDM [52].

A retrospective study of 538 patients with metastatic melanoma treated with anti-PD-1 mAbs from March 2015 to March 2018 in a single quaternary melanoma center revealed that 3 out of 10 patients with irDM were heterozygous for an HLA class II haplotype linked to increased T1DM risk, while 2 patients out of 10 patients with irDM carried HLA class II haplotypes protective against T1DM [53].

A systematic review of articles on irDM published in four databases (MEDLINE, Embase, Web of Science, and Cochrane Library) in English, between 1 January 2012 and 1 January 2018, demonstrated that an HLA genotype was correlated with an increased risk of irDM in 14 out of 21 patients subjected to HLA genotyping among a total of 42 cases of irDM [12].

In a retrospective study of the electronic medical records of 1327 adult patients who received anti-PD-(L)1 or anti-CTLA-4 mAbs from 2013 to 2018, only 1 patient out of 5 with irDM was tested for the presence of the HLA class I antigen HLA-A2 and the result was negative [54].

A systematic review and meta-analysis of papers on irDM published from 1 August 2000 to 14 August 2018 in major databases (Medline In-Process and Other Non-Indexed Citations, MEDLINE, EMBASE, Cochrane Central Register of Controlled Trials, Cochrane Database of Systematic Reviews, and Scopus) revealed that HLA typing was reported for 32 out of 71 patients with irDM. It was demonstrated that 27 out of 32 patients (85%) had at least one DR or DQ allele linked to increased T1DM risk [55].

A structured PubMed search regarding cases of irDM published from January 2015 to December 2019 identified 200 patients with irDM, of whom 49.3% were positive for HLA DR4. Although the presence of HLA haplotypes protective for conventional T1DM did not exclude the diagnosis of irT1DM, it was associated with a median time of onset of irDM significantly later than that observed in patients with other HLA haplotypes [56].

Overall, the explicit genetic susceptibility to irDM (if exists) has not been characterized yet.

#### 3.1.4. The Status of Islet Autoantibodies in the Setting of irT1DM

Islet autoantibodies are considered a hallmark of T1DM, detected in over 90% of patients with T1DM and classified in five types: islet cell autoantibodies (ICAs), insulin autoantibodies (IAAs), glutamic acid decarboxylase autoantibodies (GADAs), tyrosine phosphatase-like molecule IA-2 autoantibodies (IA-2As), and zinc transporter 8 protein autoantibodies (ZnT8As) [13,57]. The positivity of islet autoantibodies is considered, by the American Diabetes Association (ADA), as a risk factor for clinical T1DM and a potential indicator for intervention in the setting of a clinical trial [13]. Measurement of a panel of islet autoantibodies is recommended as a screening test for T1DM risk in the setting of a research trial or for first-degree family members of a proband with T1DM [13].

T1DM risk stratification models incorporating distinct characteristics of islet autoantibodies, including timing, type, and titer of positivity, may stratify the risk for T1DM more explicitly compared to the status of positivity of islet autoantibodies alone [58].

The reasons why β cells are vulnerable autoimmune targets in T1DM remain elusive. It has been postulated that intrinsic features of the biology of β cells increase their vulnerability to autoimmunity [59]. For instance, the overexpression of HLA class I molecules on β cells due to primary β-cell defects or stimulated by a trigger (e.g., a viral infection) may lead to so-called β-cell suicide. Specifically, increased β-cell endoplasmic reticulum stress may result in accelerated β-cell death, due to altered mRNA splicing and aberrant translation and folding of proteins generating potential immunogenic neoantigens [17].

So far, no direct cytotoxicity of islet autoantibodies to β cells has been proven in vitro. Additionally, islet autoantibodies may not be a prerequisite for T1DM development.

In fact, research addressing the potential association of islet autoantibodies, principally of GADA and IA-2A, with survival and residual function of β cells has yielded contradictory results [60].

Compared to the consistently high frequency of the positivity of islet autoantibodies in conventional T1DM, the positivity of islet autoantibodies in irDM is inconsistent and lower.

A review of irDM occurring over a 6-year period at two academic institutions revealed 27 patients with irDM, of whom 25 patients were subjected to measurement of at least one type of islet autoantibody, while 24 patients were subjected to measurement of three or more types of islet autoantibodies. Positivity of at least one type of islet autoantibody was found in 40% of ICPi-treated patients with irDM. Positivity of two or more types of islet autoantibodies was found in 21% of ICPi-treated patients with irDM. Positivity of one type of islet autoantibody was found in 25% of ICPi-treated patients without irDM but with cancer diagnoses similar to those of ICPi-treated patients with irDM. No patient without irDM showed positivity for more than one type of islet autoantibody. The positivity of any type of islet autoantibodies at the time of onset of irDM was associated with the onset of irDM after statistically significantly fewer ICPi cycles and after fewer weeks of ICPi treatment compared to the negativity of islet autoantibodies. The positivity of islet autoantibodies showed no correlation with the occurrence of DKA, age of patients, and body mass index (BMI) of patients. In this review, the status of autoantibodies, both before and after ICPi treatment, was investigated in three patients. The first patient presented negativity in all measured types of islet autoantibodies, both before and after treatment. The second patient presented positivity in all measured types of islet autoantibodies before ICPi treatment and positivity to only one type of measured type of islet autoantibody after ICPi treatment. The third patient presented negativity in all measured types of islet autoantibodies before ICPi treatment and positivity to three measured types of islet autoantibodies after ICPi treatment. The small number of patients and the inconsistency of findings do not allow one to draw conclusions regarding the impact of ICPis on seroconversion. Regarding self-antigens in the pancreatic islets of patients with irDM, the application of the islet cell antibody assay showed that antibody positivity concerned autoantibodies to known self-antigens in most patients [52].

A retrospective study of 538 patients with metastatic melanoma treated with anti-PD-1 mAbs from March 2015 to March 2018 in a single quaternary melanoma center revealed positivity of islet autoantibodies in 2 out of 10 patients with irDM, which exclusively concerned GADA [53].

In a review of 62 articles on irDM collected from several databases (PubMed/Web of Science/Cochrane) searched through November 2018, the incidence of islet autoantibody positivity was 51% for GADA, 18% for IA-2A, 13% for ICA, 26% for IAA, and 4% for ZnT8A in a total of 90 cases of irDM [61].

A review of 1444 patients treated with ICPi over 6 years in a cancer center revealed that 21 patients among 1163 patients treated with anti-PD-1 mAbs developed irDM. Out of these 21 patients, new-onset T1DM occurred in 12 patients. The status of islet autoantibodies was evaluated at the time of the onset of T1DM in 7 out of 12 patients and was positive in 5 out of 7 patients (71%) [62].

A retrospective study of the electronic medical records of 1327 patients who received anti-PD-(L)1 or anti-CTLA-4 mAbs from 2013 to 2018 identified 5 patients with irDM: 4 patients with new-onset T1DM and one patient with pre-existing T2DM. Two patients presented positivity to GADA, including the patient with pre-existing T2DM who also presented positivity for IAA. All patients presented negativity to ICA [54].

Analysis of 200 case reports regarding irDM published in PubMed from January 2015 to December 2019 demonstrated positivity of GADA in 43% of cases [56].

Systematic search of four databases (MEDLINE, Embase, Web of Science, and Cochrane Library) for articles regarding irDM published in English between 1 January 2012 and 1 January 2018 yielded 42 reported cases of irDM, of which 39 cases had available data for islet autoantibodies. Positivity of islet autoantibodies existed in 22 out of 39 patients (56%). GADA positivity existed in all patients, with IA-2A positivity in four patients, ICA positivity in two patients, and IAA and ZnT8A positivity in only one patient. In patients with GADA positivity, irDM was diagnosed earlier compared to patients with GADA negativity (median time of onset: 5 weeks versus 9 weeks for GADA-positive patients versus GADA-negative patients, respectively) [12].

A retrospective study investigating the characteristics of irT1DM in relation to autoantibody status and ethnic origin analyzed the data for islet autoantibodies status from 77 out of a total of 80 patients with irT1DM. Positivity of at least one type of autoantibody was observed in 20 out of 77 patients (26.0%). The frequencies of distinct types of islet autoantibodies were as follows: 21.1%, 17.5%, 8.3%, 0.04%, and 6.3% for GADA, IA-2A, ICA, and ZnT8A, respectively. The antibody-positive irT1DM group experienced an earlier time of onset of irT1DM compared to the antibody-negative irT1DM group (40 days vs. 110 days, respectively, *p* < 0.01). The number of infusions after ICPi therapy initiation was lower in the antibody-positive irT1DM group compared to that in the antibody-negative irT1DM group (three infusions vs. six infusions, *p* < 0.05). In terms of ethnicity, Caucasians with irT1DM showed significantly higher prevalence of islet autoantibodies positivity compared to Asians with irT1DM (*p* < 0.05).

A systematic review and meta-analysis on irDM through a search of major databases (Medline In-Process and Other Non-Indexed Citations, MEDLINE, EMBASE, Cochrane Central Register of Controlled Trials, Cochrane Database of Systematic Reviews, and Scopus) for papers published from August 2000 to August 2018 revealed 71 cases derived from 56 publications. Positivity of islet autoantibodies at the time of irDM presentation was observed in half of the cases. GADAs constitute the most frequently reported type of islet autoantibodies. Compared to the negativity of islet autoantibodies, the positivity of islet autoantibodies was associated with significantly earlier onset of irDM after ICPi initiation (55 days vs. 117 days, respectively; *p* = 0.005) and a higher incidence of DKA at initial presentation (86% vs. 60%, respectively; *p* = 0.02) [55].

A scoping review of case reports of endocrine irAEs based on a search of four major databases through January 2018 revealed positivity of islet autoantibodies in 51.5% of cases, negativity of islet autoantibodies in 41% of cases, and no measurement of islet autoantibodies in 7.5% of cases of irDM [63].

A brief review of case reports of DKA related to anti-PD-1 mAbs by June 2020, revealing a total of 71 cases, demonstrated positivity of one or more type of islet autoantibodies in 47% of tested cases. The most frequent types of islet autoantibodies that showed positivity were GADAs followed by IA-A2 and ZnT8A [64].

The mechanisms underlying the genesis of islet autoantibodies in irDM remain unknown. A potential mechanism is suggested from research on NOD mice, wherein the PD-1 blockade has been shown to increase IAA production de novo via an increase in T-follicular helper cells (T_FH_)/T-follicular regulatory cells (T_FR_) ratio [65], as the T_FH_ stimulate the antigen-specific humoral response while T_FR_ cells suppress B-cell activation [65,66,67,68].

Taken together, the autoimmunity of irT1DM remains an open issue and may extend beyond the islet autoantibodies–hallmark of conventional T1DM.

### 3.2. The Rationale for the Potential Involvement of the Gut Microbiome in the Pathophysiology of irT1DM

Still in its infancy, the hypothesis of the implication of the gut microbiome in the pathophysiology of irT1DM is rationalized by the emerging association of the gut microbiome, not only with T1DM but also with ICPi efficacy and safety. The gut microbiome consists of trillions of microorganisms included in all three domains of life, bacteria, eukaryotes, and archaea, as well as viruses, which colonize the human intestine, being involved in multiple gastrointestinal diseases and in extraintestinal diseases, such as metabolic, respiratory, cardiovascular, neurologic, psychiatric, autoimmune, and oncological diseases [69,70].

Accumulating evidence indicates that the gut microbiome is implicated in the development and progression of T1DM, especially in patients with genetic susceptibility to T1DM [70]. A metagenomic analysis of the gut microbiome of 74 adults with long-standing T1DM, compared to that of 296 age-matched healthy control individuals in terms of composition and function of microorganisms, revealed a discriminative microbial signature of T1DM and a correlation of several bacterial taxa and metabolic pathways with the host’s glycemic control [71]. Analysis of gut microbiome data of 238 T1DM patients with long-standing T1DM compared to that of 2937 age-, sex-, and BMI-matched controls showed that the gut microbiome of T1DM patients featured significant deficiency in 43 bacterial taxa and significant enrichment of 37 bacterial taxa. Additionally, the variability in the gut microbiome was correlated with the host’s glycemic control represented by HbA1c and diabetic complications [72].

The gut microbiome has also been credited with an immunomodulatory role, which is initiated at the intestine and eventually affects the systemic immune response. Analysis of the gut microbiome of melanoma patients treated with anti-PD-1 mAbs showed significant differences in the diversity and composition of the gut microbiome between responders and non-responders to anti-PD-1 mAbs. Compared to the gut microbiome of non-responders, the gut microbiome of responders showed higher diversity and abundance of Ruminococcaceae/Faecalibacterium, which was favorable for the host, reinforcing the antigen presentation and the effector T-cell function in both the systemic circulation and the tumor microenvironment, thereby enhancing the anticancer immune response. On the contrary, compared to the gut microbiome of responders, the gut microbiome of non-responders showed lower diversity and higher abundance of Bacteroidales. This composition in the gut microbiome in non-responders was unfavorable for the host, resulting in compromised immune response ascribed to higher levels of Tregs and myeloid-derived suppressor cells (MDSCs) in the systemic circulation, blunted cytokine secretion, decreased intratumoral lymphoid and myeloid infiltration, and decreased antigen presentation [73].

The rationale for the potential association of the gut microbiome with irT1DM is the emerging interrelationship between the gut and pancreas. Two aspects of this interrelationship have been identified: short-chain fatty acids (SCFAs) produced by the gut microbiome, which induce the secretion of cathelicidin-related antimicrobial peptide (CRAMP) by β cells, an immunoregulatory peptide inhibiting the autoimmune process of T1DM. Vice versa, oral calcium release-activated calcium modulator 1 (Orai1)-driven secretion of antimicrobial factors from pancreatic acini forms the gut microbiome. Several ongoing studies assessing the gut microbiome as a biomarker for the prognosis of cancer response to ICPis and/or for prediction of immunotoxicity are awaited to inform our understanding of the gut–pancreas crosstalk and of the potential role of the gut microbiome in irDM [70,71,72,73,74,75,76]. The dearth of strong evidence so far to sustain the hypothetical involvement of the gut microbiome in the development of irT1DM does not undermine the clinical significance of this hypothesis considering the modifiable nature of the gut microbiome.

### 3.3. The Potential Involvement of Pancreatic Alpha (α) Cells and of the Exocrine Pancreas in the Pathophysiology of irDM

The conventional β-cell-centered concept of T1DM has been revisited. Hyperglucagonemia and α-cell proliferation have been reported in patients with T1DM, induced by the dedifferentiation of β cells into α cells due to islet inflammation related to chronic hyperglycemia [77]. Additionally, several functional and morphological alterations in the exocrine pancreas have been increasingly recognized as features of T1DM, such as: (i) decreased pancreatic weight and volume [17]; (ii) immune cell infiltration and fibrosis of exocrine pancreas; (iii) deposition of C4d complement in exocrine ducts and blood vessels; (iv) decreased number of acinar cells; and (v) significantly higher CD8+ T-cell density in the exocrine pancreatic tissue [54,78]. Whether such alterations in the exocrine pancreas represent a result or, on the contrary, a cause of T1DM remains unknown. Nevertheless, there is a burgeoning effort to incorporate the levels of exocrine pancreas enzymes into T1DM risk scores for disease prediction and/or staging [78,79].

To the best of our knowledge, the involvement of α cells in irDM is underexplored. The only relevant information gained from a search of the literature in the present review comes from a case series of six patients with irDM, among whom one patient had blunted glucagon secretion [80].

The involvement of the exocrine pancreas in the pathophysiology of irDM is an interesting yet unresolved issue.

In 2018, Marchand et al. reported the first case of bihormonal pancreatic failure—irT1DM and decreased exocrine pancreatic function with acute pancreas atrophy—related to nivolumab in a 55-year-old lean Caucasian patient with metastatic lung pleiomorphic carcinoma [81]. This case report paved the way for the increasing recognition of the involvement of the exocrine pancreas in the setting of irDM.

Analysis of 200 case reports regarding irDM published in PubMed from January 2015 to December 2019 demonstrated a mild exocrine impairment in 51% of patients with irDM [56].

In a case series by Stamatouli et al., 32% of patients with irT1DM had elevated levels of lipase and/or amylase at the time of diagnosis, while in one patient, the elevation occurred one month before irDM diagnosis.

A retrospective study from Yun et al., using the electronic medical records of 1327 adult patients who received anti-PD-(L)1 or anti-CTLA-4 mAbs from 2013 to 2018, showed that among five patients with irDM, three patients had normal serum amylase levels, while two patients had modestly elevated serum amylase levels exceeding less than two-times the upper normal limits. Findings concerning the evaluation of lipase were similar to those concerning the evaluation of amylase, except for marked elevation of lipase exceeding three-times the upper normal limits in one patient. Abdominal CT scans conducted every 2–3 months to evaluate the response to treatment showed no evidence of pancreatitis [54].

The relationship between the irDM and the exocrine pancreas is further complicated by the establishment of ir pancreatitis as a distinct irAE with an incidence of 0.3–3.9% [82].

A systematic review and meta-analysis by George et al. demonstrated that treatment with anti-CTLA-4 mAbs increases the incidence of pancreatitis compared to treatment with anti-PD-1 mAbs, while an additive increase in incidence of pancreatitis is observed with a combination of anti-CTLA-4 and anti-PD-1 mAbs compared to either anti-CTLA-4 or anti-PD-1 mAbs as monotherapy [83].

A systematic review and meta-analysis by Su et al., including 15 clinical trials with 9099 patients, demonstrated that both the anti-CTLA-4 mAbs as monotherapy and the combination of nivolumab with ipilimumab increased the risk of amylase or lipase elevation compared to chemotherapy or placebo, but such regimens did not significantly increase the risk of pancreatitis compared to controls. This discrepancy highlighted the heterogeneity in the diagnostic criteria of ir pancreatitis across studies. No data concerning the development of DM in the setting of ir pancreatitis were provided in this systematic review and meta-analysis [84].

On the other hand, a review on ir pancreatitis (defined as Common Terminology Criteria for Adverse Events grade ≥ 3 lipase elevation with or without clinical symptoms) from April 2011 to April 2018 by Abu-Sbieh et al. demonstrated that 4% of ICPi-treated patients developed ir pancreatitis, of whom 7% (6 patients) developed irDM. Insulin treatment was required for five out of six patients with irDM, while the sixth patient was treated with metformin. However, no further data for the precise phenotype of irDM in these patients were provided [15].

It has been postulated that the inflammatory status of ir exocrine pancreatitis triggers immune sensitization, which, in turn, may increase the risk of islet autoimmunity, but this hypothesis is unexplored [10,85]. Overall, due to the rarity of both irDM and ir pancreatitis, the explicit mechanisms underlying ir pancreatitis, irDM, and the interrelationship thereof remain elusive.

### 3.4. The Potential Involvement of irAGL in the Pathophysiology of irDM 

The AGL is a disorder of the metabolism, with a progressive loss of subcutaneous adipose tissue throughout the whole body, principally affecting the face and the extremities [86]. Severe clinical outcomes of AGL include insulin resistance, hepatic steatosis or fatty liver, extreme hypertriglyceridemia, and chylomicronemia [86]. The AGL is often linked with various autoimmune diseases and infections causing panniculitis [86], while it has also been reported as an adverse effect of certain drugs, such as protease inhibitors [87].

The pathophysiology of AGL remains unknown but it involves autoimmunity. Indeed, autoantibodies to Perilipin-1 (PLIN1) have been identified in the sera of patients with AGL [86]. PLIN1 is a cAMP-dependent protein kinase substrate that coats the lipid storage droplets in adipocytes, protecting them from the lipolytic activity of hormone-sensitive lipase [86].

In 2019, Falcao et al. reported the first case of irAGL accompanied with irDM in a 62-year-old woman with metastatic melanoma treated with nivolumab [88]. So far, a limited number of relevant cases has been reported [88,89,90,91]. A systematic review of the literature on AGL related to anti-PD-1 mAbs based on major databases (PubMed, Embase, MEDLINE and Cochrane Central databases) revealed only four cases of AGL associated with anti-PD-1 mAbs: three associated with nivolumab and one associated with pembrolizumab. The median time of onset of irAGL was 7 months after anti-PD-1 mAbs initiation. Three out of four patients with irAGL experienced T2DM due to related insulin resistance [16].

The pathophysiology of irAGL is not completely elucidated, but, very recently, Mandel-Brehm et al. demonstrated the presence of anti-PLIN1 autoantibodies in the setting of irAGL and accompanying irT2DM in a patient with metastatic melanoma after 34 cycles of nivolumab. The irT2DM in this patient was managed with metformin, dietary changes, and high-dose basal-bolus insulin and showed improvement 4 months following cessation of nivolumab. Negativity of anti-PLIN1 autoantibodies before initiation of nivolumab was demonstrated. The seroconversion occurred after 34 cycles of treatment with nivolumab, i.e., at the time of irAGL diagnosis. The reactivity to PLIN1 decreased 19 months after cessation of nivolumab. Despite the persistence of the patient’s generalized lipodystrophy phenotype, the glycemic status in the setting of irT2DM ameliorated, requiring fewer doses of exogenous insulin [92].

## 4. The Patient-Centered Aspect of irDM

### 4.1. Pillar I: Awareness of irDM

The awareness of irDM entails knowledge of the incidence, clinical presentation, the timing, and the predictive factors of irDM [93].

The frequency of irDM is variable [8,19,52,53,54,61,62,94,95,96,97,98,99,100]. As depicted in Table 1, according to some relevant representative studies [8,19,52,53,54,61,62,94,95,96,97,98,100], the incidence of irDM ranges from 0.2% [94, 98] to 3.37% [97]. To date, it is unknown which proportion of DM is represented by irDM.

Of note, irDM is most often related to anti-PD-1/PD-L1 mAbs as compared to anti-CTLA-4 mAbs, while the risk for irDM related to combinations of anti-PD-1 or anti-PD-L1 mAbs with anti-CTLA-4 mAbs is higher compared to monotherapies [97,98]. A pharmacovigilance study revealed a marked increase in reporting irDM in the interval between 2014 and April 2018 [19]. Beyond reflecting methodological issues, the varying frequency of irDM may be ascribed, at least partially, to inconsistent recognition of irDM, highlighting the necessity for improved knowledge of the clinical presentation and timing of irDM.

The clinical presentation of irDM is multifaceted. Although irDM can be occasionally asymptomatic [12], the typical clinical manifestations of irDM are polyuria, polydipsia, weight loss, and fatigue. Alarmingly, the most common clinical presentation of irDM is DKA with a varying frequency ranging from approximately 50% to 100% [19,52,54,55,61,63,95,101,102].

In the retrospective study of Yun et al., the most common symptoms of DKA as initial presentation of irDM that forced patients to seek medical care at the hospital were fatigue, shortness of breath, confusion, blurry vision, and weight loss. In the same study, other often-reported symptoms of DKA as initial presentation of irDM were polyuria, polydipsia, abdominal pain, nausea, emesis, and diarrhea [54,56]. A rare initial presentation of irDM is the hyperosmolar hyperglycemic state, characterized by severe hyperglycemia and hyperosmolality without significant ketoacidosis, with an incidence of 1% [56].

The median age of patients who develop irDM is in the sixties, varying as follows: 61 years (range 22–84 years) in the systematic review of de Filette et al. [61], 63 years in the scoping review of Tan et al. [64], 64 years (interquartile range (IQR), 55–71) in the pharmacovigilance study of Wright et al. [19], 64.9 ± 15.2 years (minimum: 38 years; maximum: 82 years) in the observational cohort study of Rodríguez de Vera-Gómez et al. [103], 68 years in the retrospective study of Kotwal et al. [62], and 63.5 years (range 27–78 years) in the retrospective study of Byun et al. [103].

A male predominance for irDM has been consistently reported. The percentage of irDM encountered in males varies among studies [56,61,62,63,102] ranging from 57% [62] to 85.7% [102]. It remains unknown whether the male predominance for irDM reflects the male predominance to melanoma and non-small-cell lung cancer—the two most common indications for ICPi [56].

Non-Hispanic Caucasians constitute the race/ethnicity most often affected by irDM, while a percentage of 15–25% of Asians is also affected.

Melanoma and lung cancer account for the first and second, respectively, most common cancer types associated with irDM. It remains unknown whether these cancer types are most often associated with irDM because they are the most common indications for ICPi or due to a causative, yet undiscovered, link with irDM [10].

The time of onset of irDM and the number of ICPi cycles at the time of irDM diagnosis are variable across several studies, as depicted in Table 2.

Coexistence of irDM with at least one additional immune-related adverse event (irAE) and especially with an additional endocrine irAE affecting, most often, the thyroid, the pituitary, or the adrenal glands was observed in, respectively, 21% and 8.5% of cases, according to the analysis of the VigiBase—the World Health Organization’s database of individual case safety reports—from 2014 to April 2018 [19]. A review of 72 cases of irDM published in PubMed, Medline, and Google Scholar from 2004 to November 2019 demonstrated that 70% of the cases of irDM presented additional irAEs and 44% presented other endocrine irAEs, before or concurrently with irDM [105].

So far, no well-established predictive factors of irDM exist.

### 4.2. Pillar II: Diagnosis of irDM

#### 4.2.1. Screening of ICPi-Treated Patients for Hyperglycemia

To enable prompt diagnosis of irDM, the clinical practice guideline for the management of irAEs released in 2018 by the American Society of Clinical Oncology in collaboration with the National Comprehensive Cancer Network (NCCN) recommend conducting screening measurements of fasting plasma glucose at baseline and at each cycle of ICPi treatment during the first 12 weeks and then every 3 to 6 weeks [106].

The expert consensus statement on irAEs by Smati et al. [107] and the French Endocrine Society Guidance on endocrine side effects of immunotherapy by Castinetti et al. [108] recommend screening measurement of fasting plasma glucose and of HbA1c before the initiation of treatment with anti-PD-1 or anti-PD-L1 mAbs but not with anti-CTLA-4 mAbs. Additionally, patient education is crucial to recognize the initial symptoms of irDM without delay. Importantly, patients with pre-existing DM should be encouraged to initiate or reinforce the self-monitoring of glucose levels after ICPi initiation.

Baseline screening measurement of islet autoantibodies is not recommended as a routine practice because the predictive value of the positivity thereof for the development of irDM remains uncertain. However, the positivity of islet autoantibodies has been associated with an earlier onset of irDM compared to the negativity thereof [11].

#### 4.2.2. Diagnostic Work-Up of irDM

In a way reminiscent of the difficult classification of conventional DM [109], in patients receiving ICPis, any clinical suspicion of DM should prompt the measurement of plasma blood glucose and of HbA1c [110]. The diagnostic criteria for irDM are similar to those of conventional DM, as depicted in Table 3 [13]; however, the value of HbA1c may not be a reliable diagnostic criterion for irDM because it often shows disproportionately mild elevation in relation to marked hyperglycemia, indicating abrupt β-cell destruction.

In a way reminiscent of the difficult classification of conventional DM [109], the classification of irDM into irT1DM or irT2DM is not always straightforward [110]. According to the NCCN Clinical Practice Guidelines in Oncology for management of Immunotherapy-Related Toxicities, Version 1.2019, new-onset fasting glucose value < 200mg/dL, with or without history of T2DM without clinical or laboratory evidence of DKA or T1DM, is considered steroid-induced hyperglycemia or pre-existing T2DM, while new-onset fasting glucose value > 200 mg/dL or random blood glucose level > 250 mg/dL or known T2DM with fasting blood glucose level > 250 mg/dL is considered new onset irT1DM [6]. In the case of overt or suspected DKA, the initial laboratory testing should include, beyond plasma glucose and HbA1c, the evaluation of electrolytes, serum and urine ketones, complete blood count, arterial (or venous) blood gases, and acid base status [111,112].

According to the clinical practice guideline for the management of irAEs in patients treated with ICPi released in 2018 by the American Society of Clinical Oncology in collaboration with the National Comprehensive Cancer Network (NCCN), fasting glucose value > 160 mg/dL is most probably indicative of irT2DM, as opposed to fasting glucose value > 250 mg/dL, which is most probably indicative of irT1DM. Fasting glucose value > 160 mg/dL but <250 mg/dL can indicate either irT1DM or T1DM, depending on the clinical context [106].

The positivity of islet autoantibodies can confirm the diagnosis of irDM and especially of irT1DM [106,107,108], but it is not a consistent finding in irT1DM; accordingly, the negativity of islet autoantibodies cannot exclude the diagnosis of irDM [107,108]. First-line measurement of GADAs during the diagnostic evaluation of suspected irDM in ICPi-treated patients is recommended; in the case of negativity of GADAs, measurement of IA-2A and ZNT8A should be conducted [107].

A rapid decline in C-peptide levels at the initial presentation of ir hyperglycemia can confirm the diagnosis of irDM and classify irDM into irT1DM [12,61,62], even in the clinical suspicion of insulin resistance related to treatment with corticosteroids [11]. Nevertheless, inappropriately normal C-peptide levels at the initial presentation of ir hyperglycemia do not exclude the diagnosis of irDM [53,103], but they should be remeasured one month later to eliminate the effect of confounding factors, such as glucose toxicity and renal impairment [11].

It has been suggested to subclassify the irT1DM into irT1αDM and ir fulminant diabetes (FD) (irFD), the latter being reminiscent of FD, which is a subtype of conventional TIDM. FD is encountered mainly in East-Asian populations and rarely in Caucasians, featured by abrupt β-cell destruction, profound hyperglycemia, DKA despite almost normal HbA1c values, no insulin secretion in response to glucagon test, increased serum pancreatic enzyme, and close correlation with HLA class II haplotypes (DRB1*04:05–DQB1*04:01 and DRB1*09:01–DQB1*03:03) [81,113]. However, this subclassification has not been favored so far due to the distinct profile of irDM.

#### 4.2.3. Differential Diagnosis of irDM

The differential diagnosis of irDM from other cancer-related forms of hyperglycemia, irrespective of ICPis, can be daunting and involves the following conditions: (i) DM secondary to pancreatic insufficiency due to pancreatic cancer or metastases [114,115]; (ii) DM induced by anticancer treatments other than ICPis, mainly glucocorticoids, mammalian target of rapamycin (mTOR) inhibitors, 5-fluorouracil (5-FU), PI3K inhibitors, epidermal growth factor receptor (EGFR) inhibitors, multikinase inhibitors, tyrosine kinase inhibitors (TKIs), anaplastic lymphoma kinase (ALK) inhibitors, FMS-like tyrosine kinase-3 (FLT3) inhibitors, somatostatin analogues, anti-estrogen therapy, and anti-androgen therapy; and (iii) pre-existing T2DM or precipitation of the transition from pre-existing prediabetes to diabetes due to aberrant lifestyle induced by the cancer itself [116,117].

To guide the differential diagnosis of ir hyperglycemia, it is essential to scrutinize the personal history of the cancer patients with a focus on clinical and laboratory evidence about primary and metastatic tumors, exposure to drugs interfering with glucose levels, and known pre-existing T2DM or prediabetes. Unfortunately, there are no established criteria to discriminate the irT2DM that develops de novo from the irT2DM that develops after ICPi-induced precipitation of pre-existing prediabetes or from the aggravation of pre-existing T2DM, irrespective of ICPis.

**Figure 4:**
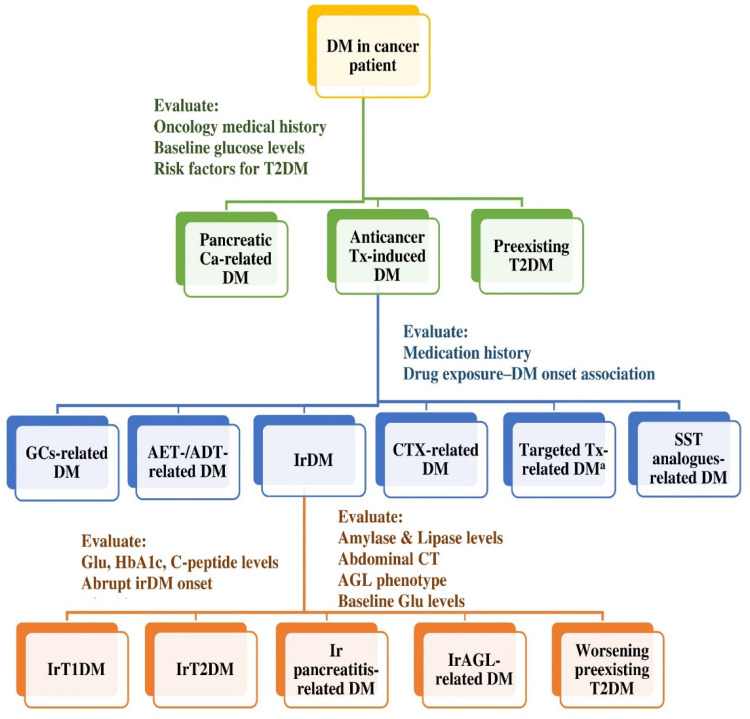
Differential diagnosis of DM and classification of irDM in cancer patients. ^a^ Targeted anticancer treatments known to cause DM include mammalian target of rapamycin (mTOR) inhibitors, phosphoinositide-3 kinase (PI3K) inhibitors, epidermal growth factor receptor (EGFR) inhibitors, multikinase inhibitors, tyrosine kinase inhibitors (TKIs), anaplastic lymphoma kinase (ALK) inhibitors, and FMS-like tyrosine kinase-3 (FLT3) inhibitors.The first step in the differential diagnosis of DM in cancer patient involves pancreatic cancer-related DM, anticancer treatment-related DM, and pre-existing DM. This step is guided by evaluation of the patient’s oncology medical history, baseline glucose levels, and DM risk factors. Once the diagnosis of anticancer treatment-induced DM is set, the second step in the differential diagnosis of DM is to identify the explicit anticancer treatment that induced DM. This step is guided by evaluation of patient’s medication history and investigation of the association of the onset of DM with exposure to a drug known to cause DM, such as GCs, AET, ADT, ICPi, CTX, targeted therapy, and SST analogues.The classification of irDM into irT1DM or irT2DM can be facilitated by an evaluation of HbA1c levels and C-peptide levels, as well as by the abrupt onset of irT1DM. The classification of irDM into ir pancreatitis-related DM, irAGL-related DM, or ICPi-induced worsening of pre-existing T2DM can be facilitated by evaluation of amylase and lipase levels, abdominal CT, presence of any AGL phenotype in the patient after ICPi initiation, and baseline glucose levels. Abbreviations: ADT, androgen deprivation therapy; AET, anti-estrogen therapy; AGL, acquired generalized lipodystrophy; Ca, cancer; CT, computed tomography; CTX, chemotherapy; DM, diabetes mellitus; GCs, glucocorticoids; Glu, glucose; ICPi, immune checkpoint inhibitors; Ir, immune related; IrDM, immune-related diabetes mellitus; IrT1DM, immune-related type 1 diabetes mellitus; IrT2DM, immune-related type 2 diabetes mellitus; SST, somatostatin; Tx, treatment.

Figure 4 illustrates an algorithm for the differential diagnosis and the classification of irDM in cancer patients.

### 4.3. Pillar III: Treatment of irDM

According to the clinical practice guideline for the management of irAEs in patients treated with ICPis released in 2018 by the American Society of Clinical Oncology in collaboration with the National Comprehensive Cancer Network (NCCN), the treatment of irDM depends on the severity of hyperglycemia [107]. In grade (G) 1 (G1) irDM (no or mild symptoms; fasting glucose value > 160 mg/dL without laboratory evidence of ketosis or T1DM), oral antidiabetic therapy is initiated for treatment of new-onset T2DM. Diagnostic evaluation for T1DM is recommended in the case of acute onset of hyperglycemia with previous normal glucose values or clinical suspicion of ketosis [106].

In G2 irDM (moderate symptoms, ability to perform activities of daily living, fasting glucose value > 160 but <250 mg/dL or ketosis or evidence of T1DM at any glucose level), insulin should be administrated for T1DM or as default therapy if the irDM type is uncertain. Hospitalization should be considered if outpatient evaluation is not feasible or in clinical suspicion of DKA [106].

In G3–4 irDM (severe symptoms, medically significant or life-threatening consequences, inability to perform activities of daily living; G3: glucose levels > 250 but <500 mg/dL; G4: glucose levels > 500 mg/dL), insulin therapy should be initiated for all patients [106].

The general principles of insulin therapy in the setting of irDM are similar to those in the setting of conventional DM. To optimize glycemic control, basal-prandial insulin regimens are preferable, comprising a long-acting insulin analogue and a short-acting insulin analogue to manage the fasting plasma glucose and the post-meal glucose peaks, respectively [106].

ICPis can be continued in G1 irDM under close clinical and laboratory evaluation, may be held in G2 irDM, and should be held in G3 irDM. ICPi treatment that has been withheld can be resumed when symptoms of hyperglycemia revert to G1 irDM [105]. An ASCO Guideline Update on the management of irAEs in patients treated with ICPi therapy, released in 2021, recapitulated the cornerstones of the management of irDM [109].

Additional expert committees have released guidelines similar to those released by ASCO, such as the expert consensus statement on irDM [107], the French Endocrine Society Guidance on endocrine side effects of ICPis [108], and the National Comprehensive Cancer Network Version 1.2019 [6].

Given that cancer patients with conventional DM have a 50% increased risk of all-cause mortality compared to cancer patients without DM [118], the management of irDM in cancer patients should not be disregarded. However, the glycemic targets in cancer patients should be individualized to accomplish a balance between patient safety and profit gained from correction of hyperglycemia. Although an HbA1c goal <7% is appropriate for many cancer patients, less or more stringent glycemic targets may be clinically indicated [119].

DKA is a medical emergency necessitating immediate hospitalization [6,105,106,107,108,110,120]. The fundamental steps of DKA management are: (i) cautious intravenous fluid resuscitation; (ii) exogenous insulin given through intravenous route initially but shifted to subcutaneous route after DKA resolution; (iii) correction of electrolyte disturbances; (iv) correction of acid–base balance [112,113].

Glucocorticoids are not indicated for the treatment of irDM due to increased risk of deterioration in blood glucose and inability to restore β-cell destruction [12,54,56,81]. No immunosuppressive treatment for irDM has been established yet.

Table 4 recapitulates the most widely applied guidelines of expert committees for the management of irDM.

### 4.4. Pillar IV: Monitoring of irDM

The monitoring of patients with irDM comprises periodical clinical evaluation and laboratory testing as clinically indicated and is of paramount clinical importance for many reasons. First, irDM is most often chronic, defined as persisting for at least 12 weeks after ICPi cessation, necessitating permanent treatment. The prevailing explanation of this chronicity considers irDM as “burnout” toxicity featuring irreversible damage, while most irAEs are considered “smouldering toxicities”, featured by off-target T-cell activation that waxes and wanes, resolving after administration of steroids or ICPi withdrawal or both interventions [10,18]. However, exceptionally rare cases of reversible irDM have been reported [104,121], rendering the monitoring essential to avert a deleterious continuation of unnecessary insulin therapy. Second, the monitoring will help to better assess the risk–reward ratio for the resumption of ICPi treatment that was withdrawn due to severe irDM, called ICPi rechallenge [122,123,124]. Third, monitoring can help to illuminate the increasingly, though not consistently, reported positive association of irAEs with ICPi efficacy [125,126,127,128,129]. Finally, monitoring will help to detect the rare, but real, ir autoimmune polyendocrine syndrome type 2 (APS-2), which is a constellation of autoimmune hypoadrenalism, thyroid dysfunction, and/or T1DM related to ICPis [130].

**Figure 5:**
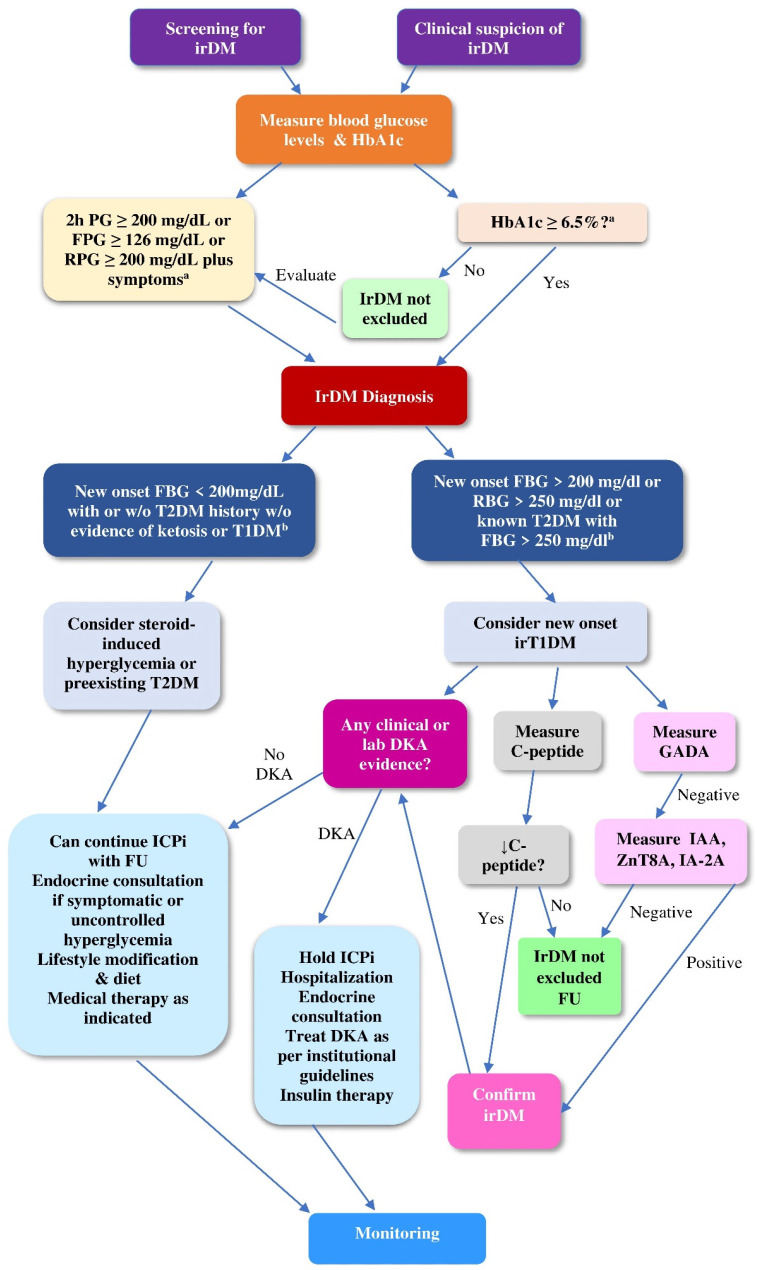
Stepwise decision making on irDM integrating the diagnostic work-up, the treatment, and the continuation or withdrawal of ICPi treatment. ^a^ According to American Diabetes Association; 2. Classification and Diagnosis of Diabetes: Standards of Medical Care in Diabetes—2020 [13]. ^b^ According to the NCCN Clinical Practice Guidelines in Oncology for management of Immunotherapy-Related Toxicities, Version 1.2019 [6] Abbreviations: DKA, diabetic ketoacidosis; FPG, fasting plasma glucose; FU, follow-up; GADAs, glutamic acid decarboxylase autoantibodies; HbA1c, glycated hemoglobulin; IAAs, insulin autoantibodies; IA-2As, tyrosine phosphatase-like molecule IA-2 autoantibodies; ICPis, immune checkpoint inhibitors; irDM, immune-related diabetes mellitus; irT1DM, immune-related type 1 diabetes mellitus; irT2DM, immune-related type 2 diabetes mellitus; lab, laboratory; Ref, reference; RPG, random plasma glucose; ZnT8As, zinc transporter 8 protein autoantibodies.

To recapitulate the patient-centered aspect of irDM, Figure 5 illustrates stepwise decision making on irDM.

## 5. Current Challenges and Future Perspectives Regarding irDM

The path forward for a concerted vision suggested to advance the knowledge of irDM is a multidisciplinary initiative to counteract current challenges and delineate future perspectives.

A major challenge as regards the understanding of the pathophysiology of irDM is to untangle the interplay between cancer biology, the biological background of the immunotoxicity, the immune profiling of ICPi-treated patients, and the biology of DM. Worldwide institutions with a great body of biomedical research and sample collections should be encouraged to create repositories of biospecimen and corresponding data with proper patients’ consent to provide material for pertinent translational research. Such translational research is expected to identify tumor-specific, patient-specific, and agent-specific factors implicated in the pathophysiology of irDM as well as modifiable and druggable underlying signaling pathways. Hopefully, such research will also illuminate the distinct autoimmune nature of irDM. Additionally, it may consolidate the postulated background of the predilection of anti-PD-1/anti-PD-L1 mAbs for the development of irDM and may illuminate the emerging, yet inconsistent, association of the CTLA-4 gene and its polymorphisms with genetic susceptibility to T1DM. If the latter association is established, it may launch a new field of research on the uncertain role of anti-CTLA-4 mAbs in the development of irT1DM [131,132,133].

To date, the only available data regarding the pathophysiology of irDM concern irT1DM. The elucidation of the pathophysiology of irT2DM is hampered by the difficulties in its differential diagnosis from pre-existing T2DM or from worsening of glycemic control, irrespective of ICPis. The exceptionally rare cases of irDM, recently reported in the setting of ir pancreatitis and ir-acquired generalized lipodystrophy (AGL) (irAGL), are not adequate to illuminate the pathophysiology of the pertinent types of irDM, except from the hypothesis that T2DM related to irAGL is ascribed to insulin resistance and implicates the presence of anti-PLN-1 antibodies, which was cited in Section 3.4 of the present review, entitled “The potential involvement of irAGL in the pathophysiology of irDM”. The cornerstone of the pathophysiology of conventional T2DM is the integration of dysregulated insulin secretion leading to chronic exposure of tissues to compensatory hyperinsulinemia with impaired insulin signaling molecular pathways causing insulin resistance [134]. Exploring whether treatment with ICPis can harm β-cell function and/or determine the metabolic switch from insulin sensitivity to insulin resistance could illuminate the pathophysiology of irT2DM. DM related to chronic pancreatitis is considered a subtype of T2DM due to insulin deficiency ascribed to compromised β-cell function caused by destruction and loss of pancreatic islets in the setting of fibrosis and parenchymal atrophy related to pancreatitis. The pathophysiology of DM in chronic pancreatitis integrates mechanisms typical of T2DM (e.g., obesity, genetic variants) with pancreas-specific mechanisms (e.g., pancreatic calcification, impairment of exocrine pancreas). Whether the aforementioned mechanisms exist in irDM related to ir pancreatitis remains to be explored. Interestingly, the development of DM related to chronic pancreatitis is strongly associated with the duration of chronic pancreatitis, mirroring progressive dysfunction and destruction of β cells [135]. In that respect, given that ir pancreatitis typically presents as acute pancreatitis, potentially evolving to chronic pancreatitis, it would be of interest to investigate the natural history of irDM related to ir pancreatitis, which may reveal any distinct underlying mechanisms [15].

The refinement of all pillars of the patient-centered aspect of irDM—awareness, diagnosis, treatment, and monitoring—can be facilitated by improved understanding of the pathophysiology of irDM, but it extends beyond that.

With respect to the awareness of irDM, a major challenge is to predict irDM. To counteract this challenge, in-depth understanding of the pathophysiology of irDM is expected to provide molecular, genomic, epigenetic, and immunological predictive biomarkers, the integration of which will enable the prediction of irDM and the development of stratification models for ICPi-treated patients according to personalized risk for irDM.

Several potential biomarkers for predicting the development of irAEs are currently under exploration [122,136,137]. Investigation of peripheral molecular predictive biomarkers of irDM is preferable due to easy accessibility of peripheral blood as opposed to technical obstacles to tumor sampling. Current challenges in the validation of circulatory biomarkers—i.e., variability in assays, platforms, and reference standards—could be counteracted via harmonization and standardization of key relevant platforms [138].

Potential predictive tools for irDM that merit further evaluation are: (i) the profile of microRNAs (mRNAs) known to regulate gene expression involved in β-cell death [139]; (ii) the phenotyping of T cells before ICPi initiation, as indicated by the association of the pre-existing effector insulin-specific CD4+ T cells with increased risk of irDM after PD-1 blockade in mice [65]; (iii) the integration of involved gene expression and/or multi-omics data with other pertinent risk factors [140]; (iv) the positivity of status of islet autoantibodies, if a consistent and causative association thereof with irDM is established; (v) the baseline profiles of autoantibodies, other than islet autoantibodies, that are differentially expressed in ICPi-treated patients and have been shown to predict immunotoxicity [141,142,143,144,145,146]; (vi) the polygenic risk scores [10,49,144], incorporating but not being limited to variants in the MHC locus and/or the PD-1/PD-L1 locus [10,145].

Importantly, an improved understanding of the autoimmune background of the pathophysiology of irDM may help to assess the risk for irDM in patients with pre-existing autoimmune disease (AID) [137,143,146].

A major challenge arising from the classification and the differential diagnosis of irDM, hampering the explicit diagnosis of irDM, is the lack of established criteria to classify irDM into T1DM or T2DM and/or to discriminate the irT2DM that develops de novo from the ICPi-induced precipitation of pre-existing prediabetes or from the aggravation of pre-existing T2DM, irrespective of ICPis. In-depth understanding of the pathophysiology of irDM may yield classification models integrating clinical features, molecular biomarkers, islet autoantibodies, and irT1DM genetic scores to be validated in clinical practice [28].

Beyond the exploitation of the understanding of the pathophysiology of irDM, additional strategies to further refine the awareness and the diagnosis of irDM should aim to better characterize the epidemiological and clinical profile of irDM. To this end, it is necessary to counteract the following challenges: (i) variance in reporting terms for irDM across trials; (ii) absence of universal recording systems; (iii) heterogeneity in the policy of screening; (iv) differences among studies regarding ICPi classes and subclasses; (v) shortfalls in patient recruitment; (vi) differences in terms of whether the irAEs are primary or secondary endpoints; and (vi) selection bias due to exclusion of certain categories of patients, such as patients with AID from clinical trials. A prerequisite for overcoming these challenges is the adoption of coherent universal strategies directed towards: (i) a consensus on a comprehensive list of terms used to describe irDM; (ii) the standardization of reporting algorithms for irDM; (iii) the development of protocols for prospective and retrospective collection of data on irDM; (v) the development of electronic health records accessible to involved health-care providers; (vi) conducting well-designed prospective randomized controlled clinical trials on irDM with a focus on subgroup of patients with pre-existing comorbidities, such as autoimmune diseases (AIDs), cardiac disease, organ transplantation, liver dysfunction, kidney dysfunction, and allogeneic stem cell transplantation [137].

Given the rarity of irDM, exploitation of global registries through implementation of artificial intelligence techniques and embracement of the new Side Effect Reporting Immuno-Oncology (SERIO) recommendations can help to explicitly estimate the incidence of irDM [147].

Considering the unpredictable time of onset of irDM, long-term follow-up of ICPi-treated patients and quantification of the risk for irDM are needed to assess the cumulative incidence of irDM, defined as the probability of occurrence over time [148].

As regards the treatment of irDM, a major challenge is the lack of antihyperglycemic treatments for irDM other than the known antidiabetic medications. In-depth understanding of the pathophysiology of irDM may yield future therapeutic perspectives, namely: (i) the empowerment of a so-called shut-off strategy to inhibit key inflammatory processes with limited immunosuppressive impact on the response of tumor to ICPi [149,150] through innovative immunosuppressive agents, such as vedolizumab (anti-integrin α4β7), infliximab (a chimeric monoclonal anti-tumor necrosis factor (TNF) alpha (TNF-α) antibody), tocilizumab (anti-interleukin (IL)-6 (IL-6) receptor antibody), mycophenolate mofetil, cyclophosphamide, and intravenous immunoglobulins [10]; (ii) the use of breakthrough modalities of regenerative medicine, such as the pancreatic islet neogenesis-associated protein (INGAP), which induces the neogenesis of the native endocrine pancreas [151,152]; (iii) the modulation of the gut microbiome through diet, probiotics, prebiotics, synbiotics, postbiotics, and fecal microbiota transplantation (FMT) to create a composition capable of protecting the host against irDM [70,106].

An additional challenge arising from the treatment of irDM is the potential interference of certain antidiabetic drugs with the efficacy of ICPis. For instance, mouse data have shown that the efficacy of anti-PD-1 mAbs is reinforced by acarbose and sitagliptin, unaffected by metformin and subverted by glimepiride, pioglitazone, and insulin [153]. Further investigation of drug–drug interactions is needed to facilitate the proper selection of antidiabetic drugs for ICPi-treated patients.

The major challenges regarding the monitoring of irDM are: (i) the elusive natural history of irDM [10,18]; (ii) the uncertainty about recurrence of irDM after ICPi rechallenge [122,123,124]; and (iii) the emerging association of irDM with ICPi efficacy [10,18]. Delving into the pathophysiology of irDM could counteract the aforementioned challenges and delineate the following future perspectives: (i) identification of the potential determinants of the chronicity or on the contrary the reversibility of irDM; (ii) identification of the potential determinants of the recurrence of irDM after ICPi rechallenge; (iii) clarification of the determinants of the association of irDM with the therapeutic efficacy of ICPi; and (iv) development of strategies to disconnect the response to ICPi from immunotoxicity, such as administration of ICPi directly within the tumor microenvironment [154] and bispecific [155] or multipiece antibody-based strategies [156]. Whether the association of DM that is unrelated to ICPi with decreased ICPi efficacy [157] and increased incidence and/or unfavorable prognosis of certain cancer types [158] can be translated into an unfavorable impact of irDM on ICPi efficacy and cancer prognosis needs to be further addressed.

Additionally, the exploitation of global registries and databases and the long-term follow-up of patients with irDM may inform the understanding of the natural history of irDM. Finally, the conduction of well-designed prospective clinical studies will help to evaluate the association of irDM with ICPi efficacy. Table 5 summarizes the current challenges and future perspectives concerning irDM. Overall, the scientific-centered aspect of irDM can inform the patient-centered aspect decision making on irDM, while, vice versa, the latter can provide clues for translational research to advance the former.

## 6. Conclusions

**Figure 6:**
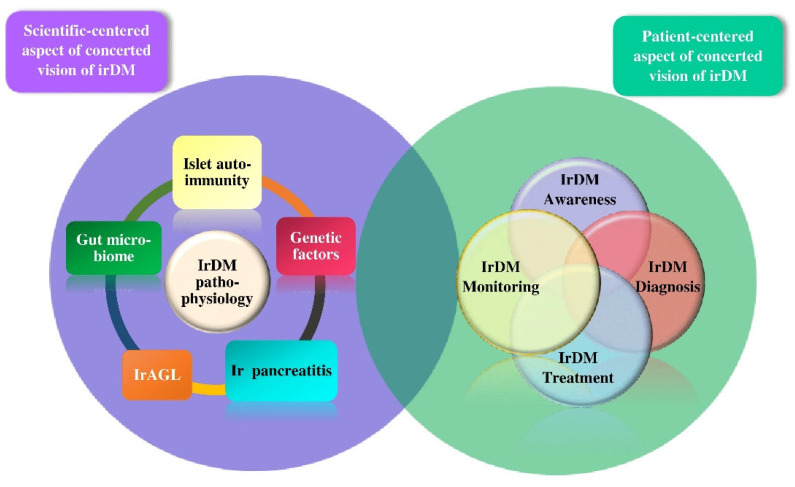
The concerted vision to advance the knowledge of irDM integrating 2 intertwined aspects: a scientific-centered and a patient-centered aspect. Abbreviations: irAGL, immune-related acquired generalized lipodystrophy; irDM, immune-related diabetes mellitus.

IrDM is conceived as an umbrella term encompassing various forms of hyperglycemia related to ICPis, among which irT1DM prevails—a distinct entity presenting similarities with, but also differences from, conventional T1DM. To advance the knowledge of irDM, the present review suggests a concerted vision comprising two intertwined aspects: a scientific-centered and a patient-centered view. The scientific-centered aspect addresses the pathophysiology of irDM, involving islet autoimmunity, genetic factors, the gut microbiome, ir pancreatitis, and irAGL. The patient-centered aspect integrates four interrelated clinical pillars: awareness, diagnosis, treatment, and monitoring of irDM. This concerted vision is illustrated in Figure 6.

Albeit not well-established, the prevailing hypothesis for the pathophysiology of irT1DM delineates the interplay between a disinhibition of PD-1/PD-L1 interaction, islet autoantibodies, and genetic susceptibility. The role of the gut microbiome, ir pancreatitis, and irAGL in the pathophysiology of irDM is yet to be unveiled. Pending more high-quality evidence on the management of irDM, clinicians’ judgement is seminal in the patient-centered aspect. Increased awareness will enable prompt recognition of irDM and referral of patients to endocrinologists to avert a life-threatening DKA. The decision-making is guided by the severity of hyperglycemia. The cornerstone of the treatment of mild or severe hyperglycemia related to ICPis is insulin. The monitoring of irDM is critical to inform the understanding of the natural history of irDM, ensure the safety of ICPi rechallenge, and assess the prognostic value of irDM for the efficacy of ICPis. The path forward for the concerted vision for irDM is a multidisciplinary initiative oriented towards: (i) improved characterization of the epidemiological, clinical, and immunological profile of irDM; (ii) exploitation of global registries; (iii) establishment of standardized protocols for diagnosis, management, and monitoring of irDM; (iv) stratification of ICPi-treated patients according to personalized risk for irDM; (v) new therapeutic options for irDM; and (vi) disconnection of ICPi efficacy from irDM.

## Figures and Tables

**Table 1:** Representative studies regarding the frequency of irDM.

Study Type [Ref]	Methods/Patients	Results
Systematic review [8]	Search of PubMed through 22 August 2017 regarding endocrine irAEs yielded 101early phase I/II, phase III experimental trials, prospective and retrospective observational studies, comprising 19,922 patients.	Incidence of irDM related to nivolumab: 2.0% (95% CI, 0.7–5.8). Incidence of irDM related to pembrolizumab: 0.4% (95% CI, 0.2–1.3). IrDM was principally related to anti-PD-1/PD-L1 mAbs. No case of irDM was related to anti-CTLA-4 mAbs.
Pharmaco-vigilance study [19]	Pharmacovigilance analysis of the VigiBase from 2014 to April 2018 revealed 283 cases of irT1DM	Most cases of irT1DM were encountered in patients receiving anti-PD-1 monotherapy of whom 52.7% received nivolumab and 23.3% received pembrolizumab. Only 12 cases of irT1DM were related to ipilimumab monotherapy. Notable increase in irDM reporting from 2014 to April 2018: ✓ only 1% of all cases was reported in 2014. ✓ over 50% of all cases were reported in 2017.
Retrospective study [52]	Ρeview of cases of irDM occurring over a 6-year period (2012–2018) at two academic institutions yielded 27 patients with irDM.	IrDM incidence: 0.9%. Patients with irDM had various solid-organ cancer types, all treated with anti-PD-1 mAbs or anti-PD-L1 mAbs or combination of ipilimumab with nivolumab. No irDM case was related to anti–CTLA-4 mAbs as monotherapy.
Retrospective study [53]	Study of 538 patients with metastatic melanoma treated with anti-PD-1-based immunotherapy from March 2015 to March 2018 in a single quaternary melanoma center	IrDM incidence: 1.9%. Among 10 patients with irDM, only one patient had pre-existing T2DM.
Retrospective study [54]	Review of electronic medical record of 1327 adult patients who received anti-PD-(L)1 or anti-CTLA-4 mAbs from 2013 to 2018.	IrDM incidence: 0.38% (5 patients). All irDM cases were related to anti-PD-1 mAbs. 2 irDM cases were related to combination of anti-PD-1 mAb with ipilimumab
Case report of irDKA and systematic review [61]	Search of PubMed, Web of Science, and Cochrane, through November 2018 for cases of autoimmune DM related to ICPi yielded 90 irDM cases.	Anti-PD-1 monotherapy was responsible for 71% of irDM cases. Anti-PD-1/PD-L mAbs were responsible for 96% of all irDM cases. IrDM related to anti-CTLA-4 mAbs comprised only 3% of irDM. All anti-CTLA-4 mAbs-treated patients who presented irDM were pre-treated with nivolumab and/or interferon.
Retrospective study [62]	Review of 1444 patients treated with ICPi in a single center from January 2012 to December 2017 revealed 1163 patients treated with anti-PD-1 among whom 21 patients developed irDM	Out of 21 patients with irDM: ✓ 12 patients experienced new-onset insulin-dependent irDM (frequency: 1%). ✓ 9 patients experienced deterioration of pre-existing T2DM (frequency: 0.8%). Among the pembrolizumab-treated patients, 2.2% met criteria for irDM, of which: ✓ 1.4% had new-onset insulin-dependent irDM. ✓ 0.8% had worsening of pre-existing T2DM. Among the nivolumab-treated patients, 1% met criteria for irDM, of which: ✓ 0.2% had new-onset insulin-dependent irDM. ✓ 0.8% had worsening of pre-existing T2DM. No cases of irDM were related to anti-CTLA-4 mAb (ipilimumab) alone.
Systematic review and meta-analysis [94]	Search of PubMed through July 18, 2016 regarding endocrine irAEs yielded 38 randomized clinical trials comprising 7551 patients eligible for a meta-analysis.	Incidence of insulin dependent DM of any-grade: 0.2% (13 cases). Incidence of insulin dependent G ≥ 3 DM: 0.1% (6 cases). All cases of insulin dependent DM except one were related to anti-PD-1 mAbs.
Retrospective study [95]	Search of PubMed between January 2016 and April 2018 regarding irAEs yielded 101 publications, which reported 139 cases of irAEs.	irDM was the most common endocrine irAE. Among 54 reported cases of endocrine irAEs, there were 22 cases of irDM, of which: ✓ 4 related to pembrolizumab. ✓ 3 related to combination pembrolizumab/ipilimumab. ✓ 11 related to nivolumab. ✓ 4 related to combination nivolumab/ipilimumab.
Systematic review and meta-analysis [96]	Search of PubMed, Cochrane library, Web of Science, and ClinicalTrials.gov between January 1990 and March 2018 regarding studies reporting irAEs related to anti-CTLA-4 mAbs yielded a total of 11 clinical trials with 7088 patients of whom 10 clinical trials had data accessible on ClinicalTrials.gov.	No case of irDM related to anti-CTLA-4 mAb was identified
Meta-analysis [97]	Search of PubMed, EMBASE, Cochrane Library databases, and ClinicalTrials.gov from the establishment of ICPi to March 2019 for RCTs regarding irDM yielded 40 trials reporting at least one irDM event, comprising 24,596 patients.	Rates of all-grade and serious-grade hyperglycemia events 2.26% (95% CI, 1.28 to 3.48) and 0.28% (95% CI, 0.16 to 0.42), respectively. Among patients receiving ICPi combination, the rates of all-grade hyperglycemia and of serious-grade hyperglycemia events were 3.37% and 0.47%, respectively. Compared to patients treated with other regimens, patients treated with ICPi were at higher risk for: ✓ serious-grade hyperglycemia (OR, 2.41; 95% CI, 1.52 to 3.82). ✓ DM (OR, 3.54; 95% CI, 1.32 to 9.51). ✓ all-grade T1DM (OR, 6.60; 95% CI, 2.51 to 17.30). ✓ serious-grade T1DM (OR, 6.50; 95% CI, 2.32 to 18.17). ICPi demonstrated a trend toward: ✓ elevated risk of all-grade hyperglycemia (OR, 1.38; 95% CI, 1.15 to 1.66). ✓ no elevated risk of T2DM (OR, 0.92; 95% CI, 0.24 to 3.52). IrDM risk in ICPi-treated patients was higher compared to control groups (excluding the control group treated with everolimus) with: ✓ OR, 4.42, 1.75, and 2.81 for DM, all-grade hyperglycemia, and serious-grade hyperglycemia, respectively. Subgroup analysis by ICPi type showed elevated risk of hyperglycemic when anti-PD-1 or anti-PD-L1 mAb was combined with anti-CTLA-4 mAb, compared to monotherapies: ✓ OR 7.35, 2.51, and 4.18 for DM, all-grade hyperglycemia, and serious-grade hyperglycemia, respectively.
Pharmaco-vigilance study [98]	Detection of signals of irAEs using the US Food and Drug Administration (FDA) adverse events (AEs) Reporting System (FAERS) database from the respective FDA approval dates for each specific drug through the second quarter of 2017.	Ir T1DM was observed in approximately 0.2% of cases.
Pharmaco-vigilance study [100]	Review of Optum’s Clinformatics Data Mart database to assess T1DM incidence and characteristics in a large de-identified cohort of ICPi-treated patients between 2017 and 2020, encompassing 30,337 patients	IrT1DM incidence: 0.86% (261/30,337 patients). Combination of anti-CTLA-4 and anti- PD-1 or anti-PD-L1 was associated with increasing risk of irT1DM (HR 1.62; 95% CI 1.15–2.26) vs. anti-PD-L1 or anti-PD-1 alone.

Abbreviations: anti-CTLA-4, antibodies against cytotoxic T-lymphocyte antigen 4 (CTLA-4); anti-PD-1,antibodies against programmed cell death (PD) 1 (PD-1); anti-PD-L1, antibodies against the ligand of PD-1 (PD-L1); CI, confidence interval; DM, diabetes mellitus; HR, hazard ratio; mAb(s), monoclonal antibody(antibodies); ICPis, immune checkpoint inhibitors; irDM, immune-related DM; irT1DM, immune-related type 1 DM; irT2DM, immune-related type 2 DM; OR, odds ratio; Ref, reference; RCTs, randomized clinical trials; vs., versus.

**Table 2:** Representative data regarding the time of onset of irDM and the number of ICPi cycles at the time of irDM diagnosis.

Ref	Range of Time Interval between ICPi Initiation and irDM Diagnosis	Median Onset Time of irDM Diagnosis	Number of ICPi Cycles at the Time of irDM Diagnosis
[12]	1 to 52 weeks	NA	Median: 3 cycles Range: 1 to 17 cycles
[19]	5 to 790 days	116 days	Median: 3 cycles Range: 1 to 24 cycles.
[53]	IQR: 17.5 to 34.5 weeks.	25 weeks	NA
[54]	20 to 972 days	NA	NA
[55]	5 to 448 days	NA	NA
[63]	NA	7.5 weeks	NA
[102]	3.6 to 45 weeks	8.14 weeks	Mean: 4.3 (SD: 2.6) Median: 3 cycles Range: 2 to 8 cycles.
[104]	0 to 122 weeks	12 weeks	NA

Abbreviations: ICPi, immune checkpoint inhibitor; IQR, Interquartile range; irDM, immune-related diabetes mellitus; NA, not applicable; Ref, reference; SD, standard deviation.

**Table 3:** The diagnostic criteria of DM.

Parameter	Diagnosis	
	Prediabetes	Diabetes Mellitus
FPG ^a^	IFG: 100–125 mg/dL (5.6–6.9 mmol/L) AND/OR	≥126 mg/dL (7.0 mmol/L). OR
2-h PG ^b^	IGT: 140–199 mg/dL (7.8–11.0 mmol/L) AND/OR	≥200 mg/dL (11.1 mmol/L) during OGTT. OR
HbA1c ^c^	5.7–6.4% (39–47 mmol/mol) or ≥10% increase in HbA1C	≥6.5% (48 mmol/mol). OR
RPG	NA	≥200 mg/dL (11.1 mmol/L) plus symptoms (polyuria, polyuria, polydipsia, weight loss, fatigue)

^a^ Fasting is defined as no caloric intake for at least 8 h. ^b^ PG measured 2 h after OGTT test performed as described by WHO, using a glucose load containing the equivalent of 75 g anhydrous glucose dissolved in water. ^c^ HbA1c is not valid as diagnostic criterion in conditions known to affect the relationship of HbA1c with glycemia, such as hemoglobinopathies, HIV, hemodialysis, recent blood loss or transfusion, or erythropoietin therapy. Additionally, HbA1c may not be reliable diagnostic criterion for the diagnosis of irDM because it may be inappropriately normal or mildly elevated despite profound hyperglycemia. FPG, 2-h PG during 75-g OGTT, and HbA1c have equal relevance for diagnosis of conventional DM. However, the plasma blood glucose criteria are preferable for the diagnosis of irDM. In the absence of clear clinical diagnosis of conventional DM, two abnormal diagnostic test results from the same sample or from two separate samples are required. In case of discordant results from two different tests, the test with abnormal result should be repeated, considering the possibility of HbA1c assay interference. The diagnosis of conventional DM is set by the confirmed test. Abbreviations: DM, diabetes mellitus; FPG, fasting plasma glucose; h, hours; HbA1c, glycated hemoglobin; IFG, impaired fasting glucose; IGT, impaired glucose tolerance; irDM, immune-related DM; NA, not applicable; OGTT, oral glucose tolerance test; PG, plasma glucose; 2-h PG, 2-h plasma glucose; RPG, random plasma glucose; WHO, World Health Organization.

**Table 4:** The main guidelines for irDM treatment by expert committees.

Expert Committees’ Guidelines for irDM Treatment				
(Year of Release)				
[Ref]				
Clinical practice guideline for the management of irAEs in patients treated with ICPi by ASCO in collaboration with NCCN (2018) [106]	Management of irAEs in Patients Treated with Immune Checkpoint Inhibitor Therapy: ASCO Guideline Update (2021) [110]	Management of Immunotherapy-Related Toxicities, Version 1.2019, NCCN Clinical Practice Guidelines in Oncology. (2019) [6]	French Endocrine Society Guidance on endocrine side effects of immunotherapy. (2019) [108]	Expert opinion on immunotherapy induced diabetes (2018) [107]
G1: Oral therapy for new-onset T2DM. Screen for T1DM if indicated. ICPi can be continued with close clinical and laboratory FU. G2: ● Oral therapy or add-on insulin therapy for worsening T2DM. ● Insulin therapy in T1DM or as default therapy. ● Hospitalization: ✓ T1DM ✓ No availability of early outpatient evaluation. ✓ Signs of DKA. May hold ICPi until Glu control. G3–4: ● Urgent endocrine consultation and insulin therapy for all patients. ● Hospitalization for: ✓ DK ✓ Symptomatic patients regardless of irDM type. ✓ New-onset T1DM without access to endocrine consultation. Should hold ICPi until reduction of toxicity to G1 or less.	● Insulin therapy as default treatment in severe hyperglycemia. ● Endocrine consultation in all cases of ir DM. ● Patients’ education for recognition of initial symptoms of ir DM. ● Hospitalization: ✓ Overt DKA. ✓ Concern for DKA. ✓ New-onset T1DM with no availability of outpatient endocrinological consultation. No immuno-suppressive treatments.	● New onset FBG < 200 mg/dL with or without T2DM history without clinical or laboratory evidence of ketosis or T1DM: ✓ Medical therapy under endocrine consultation. ✓ Diet and lifestyle modification. ✓ ICPi can be continued with FU. ● New onset FBG > 200 mg/dL or RBG > 250 mg/dL or known T2DM with FBG > 250 mg/dL: ❖ If no DKA: ✓ manage as above. ❖ If DKA: ✓ Hospitalization ✓ Institutional guidelines for management of DKA. ✓ Withhold ICPi. ✓ Tailored insulin treatment after DKA resolution.	● In view of fulminant DM: ✓ 1st line urgent initiation of insulin therapy (complex scheme). ✓ Patients’ education under endocrine consultation. ✓ HbA1c target: <8.0%. ✓ No alternative treatments.	● Insulin treatment and patients’ education in specialized endocrinology/ diabetology department. ● Regular FU and diabetology consultation. ● HbA1c target < 8.0%. ● No indication for corticosteroids. ● In view of fulminant DM with severe insulinopenia: ✓ 1st line multi-injection insulin emergency therapy in specialized center or by mobile diabetology team. ✓ No alternative treatment options. ✓ Withhold ICPi for severe irDM.

Gl: Asymptomatic or mild symptoms; fasting glucose value > 160 mg/dL without evidence of ketosis or laboratory evidence of T1DM. G2: Moderate symptoms, capability to perform activities of daily living, fasting glucose value > 160–250 mg/dL; fasting glucose value > 8.9–13.9 mmol/L, ketosis or evidence of T1DM at any glucose level. G3–4: Severe symptoms or life- threatening consequences, unable to perform activities of daily living; G3: > 250–500 mg/dL (>13.9–27.8 mmol/L); G4: > 500 mg/dL (>27.8 mmol/L). Abbreviations: ASCO, American Society of Clinical Oncology; DM, diabetes mellitus; DKA, diabetic ketoacidosis; FBG, fasting blood glucose; FU, follow-up; G, grade; Glu, glucose; HbA1c, glycated hemoglobulin; irAEs, immune-related adverse events; irDM, immune-related DM; ICPi, immune checkpoint inhibitor; NCCN, National Comprehensive Cancer Network; RBG, random blood glucose; Ref, reference; T1DM, type 1 DM; T2DM, type 2 DM.

**Table 5:** Main challenges and future perspectives regarding irDM.

Challenge	Future Perspective
Scientific-centered aspect of concerted vision for irDM	
Understanding the pathophysiology of irDM	
➢ Elusive interplay between cancer biology, immunotoxicity, immune profiling of ICPi-treated patients, and DM biology.	Establishment and exploitation of worldwide repositories of biospecimens and corresponding data for translational research on irDM. Identification of tumor-specific, patient-specific, and agent-specific molecular and/or genetic patterns of susceptibility to irDM. Identification of modifiable and/or druggable signaling pathways implicated in the pathophysiology of irDM. Explanation of the predilection of anti-PD-1/anti-PDL-1 mAbs for the irDM. Clarification of the potential association of the CTLA-4 gene with susceptibility to irT1DM. Clarification of the autoimmune nature of irDM.
Patient-centered aspect of concerted vision for irDM	
Awareness and Diagnosis	
➢ Lack of reliable predictive biomarkers to assess the risk for irDM.	Identification and validation of potential predictive biomarkers for irDM, such as: ✓ microRNAs ✓ baseline phenotyping of T cells ✓ autoantibodies signatures ✓ polygenic risk scores Development and validation of stratification models integrating molecular, genomic, epigenetic, and immunological predictive biomarkers for irDM
➢ Not straightforward classification of irDM in irT1DM and irT2DM. ➢ No established criteria to discriminate irT2DM that develops de novo from the T2DM due to ir precipitation of pre-existing prediabetes or from the aggravation of pre-existing T2DM irrespectively of ICPi.	Development and validation of models for classification and/or differential diagnosis of irDM, integrating clinical features, molecular biomarkers, islet autoantibodies, and irT1DM genetic scores.
➢ Lack of universal reporting terms for irDM. ➢ Lack of universal definitions of irDM. ➢ Methodological differences among studies. ➢ Inconsistent data regarding the status of islet autoantibodies in the setting of irDM. ➢ Inconclusive data on C-peptide levels in the setting of irDM. ➢ Limited baseline data concerning the status of blood glucose and of islet autoantibodies before ICPi initiation. ➢ Unpredictable onset of irDM. ➢ Selection bias due to exclusion of certain categories of patients, such as patients with AID, from clinical trials.	Consensus on a comprehensive list of definition terms for irDM. Standardized reporting algorithms for irDM. Establishment of protocols for prospective and retrospective collection of data on irDM. Establishment of electronic health records accessible to involved health care providers. Exploitation of global registries. Long-term FU to assess the cumulative incidence of irDM. Well-designed large-scale prospective clinical trials assessing the risk for irDM in patients with AID or other comorbidities.
Treatment	
➢ Lack of antihyperglycemic treatments for irDM other than the known antidiabetic medications. ➢ Potential interference of certain antidiabetic medications with ICPi efficacy.	Innovative immunosuppressive agents. Breakthrough pancreatic regenerative medicine to restore β cell function. Modulation of the gut microbiome through diet, probiotics, prebiotics, synbiotics, postbiotics, and FMT. Drug-drug interactions studies to facilitate appropriate selection of antidiabetic agents in ICPi-treated patients
Monitoring	
➢ Which is the natural history of irDM? ➢ How to decide about rechallenge with ICPi after irDM amelioration or reversal? ➢ Can irDM affect the ICPi efficacy?	Establishment of the natural history of irDM. Identification and modification of potential determinants of irDM reversibility. Assessment of risk of recurrence of irDM after ICPi rechallenge. Clarification of the association of irDM with the therapeutic efficacy of ICPi. Development of strategies to disconnect the response to ICPi from the immunotoxicity.

Abbreviations: anti-PD-1, antibodies against the programmed cell death (PD) 1 (PD-1); anti-PD-L1, antibodies against the ligand of PD-1 (PD-L1); AID, autoimmune diseases; CTLA-4, cytotoxic T-lymphocyte antigen 4; DM, diabetes mellitus; FMT, fecal microbiota transplantation; ICPi, immune checkpoint inhibitor; ir, immune-related; irDM, immune-related DM; irT1DM, immune-related type 1 DM; irT2DM, immune-related type 2 DM.

## Data Availability

Not applicable.

## References

[1] Shiravand Y., Khodadadi F., Kashani S.M.A., Hosseini-Fard S.R., Hosseini S., Sadeghirad H., Ladwa R., O’Byrne K., Kulasinghe A. (2022). Immune Checkpoint Inhibitors in Cancer Therapy. Curr. Oncol..

[2] Schnell A., Bod L., Madi A., Kuchroo V.K. (2020). The yin and yang of co-inhibitory receptors: Toward anti-tumor immunity without autoimmunity. Cell Res..

[3] Sharpe A., Pauken K. (2018). The diverse functions of the PD1 inhibitory pathway. Nat. Rev. Immunol..

[4] Ghosh C., Luong G., Sun Y. (2021). A snapshot of the PD-1/PD-L1 pathway. J. Cancer.

[5] Cancer Research Institute (CRI) (2023). FDA Approval Timeline of Active Immunotherapies. https://www.cancerresearch.org/fda-approval-timeline-of-active-immunotherapies.

[6] Thompson J.A., Schneider B.J., Brahmer J., Andrews S., Armand P., Bhatia S., Budde L.E., Costa L., Davies M., Dunnington D. (2019). Management of Immunotherapy-Related Toxicities, Version 1.2019, NCCN Clinical Practice Guidelines in Oncology. J. Natl. Compr. Canc. Netw..

[7] Raschi E., Mazzarella A., Antonazzo I.C., Bendinelli N., Forcesi E., Tuccori M., Moretti U., Poluzzi E., De Ponti F. (2019). Toxicities with Immune Checkpoint Inhibitors: Emerging Priorities from Disproportionality Analysis of the FDA Adverse Event Reporting System. Target Oncol..

[8] de Filette J., Andreescu C.E., Cools F., Bravenboer B., Velkeniers B. (2019). A Systematic Review and Meta-Analysis of Endocrine-Related Adverse Events Associated with Immune Checkpoint Inhibitors. Horm. Metab. Res..

[9] Deligiorgi M.V., Panayiotidis M.I., Trafalis D.T. (2020). Endocrine adverse events related with immune checkpoint inhibitors: An update for clinicians. Immunotherapy.

[10] Quandt Z., Young A., Anderson M. (2020). Immune checkpoint inhibitor diabetes mellitus: A novel form of autoimmune diabetes. Clin. Exp. Immunol..

[11] Wu L., Tsang V.H.M., Sasson S.C., Menzies A.M., Carlino M.S., Brown D.A., Clifton-Bligh R., Gunton J.E. (2021). Unravelling Checkpoint Inhibitor Associated Autoimmune Diabetes: From Bench to Bedside. Front. Endocrinol..

[12] Clotman K., Janssens K., Specenier P., Weets I., De Block C.E.M. (2018). Programmed Cell Death-1 Inhibitor-Induced Type 1 Diabetes Mellitus. J. Clin. Endocrinol. Metab..

[13] American Diabetes Association (2020). 2. Classification and Diagnosis of Diabetes. Standards of Medical Care in Diabetes—2020. Diabetes Care.

[14] Sayed Ahmed A., Abreo M., Thomas A., Chari S.T. (2022). Type 3 autoimmune pancreatitis (immune checkpoint inhibitor-induced pancreatitis). Curr. Opin. Gastroenterol..

[15] Abu-Sbeih H., Tang T., Lu Y., Thirumurthi S., Altan M., Jazaeri A.A., Dadu R., Coronel E., Wang Y. (2019). Clinical characteristics and outcomes of immune checkpoint inhibitor-induced pancreatic injury. J. Immunother. Cancer.

[16] Gnanendran S.S., Miller J.A., Archer C.A., Jain S.V., Hwang S.J.E., Peters G., Miller A. (2020). Acquired lipodystrophy associated with immune checkpoint inhibitors. Melanoma Res..

[17] DiMeglio L.A., Evans-Molina C., Oram R.A. (2018). Type 1 diabetes. Lancet.

[18] Johnson D.B., Nebhan C.A., Moslehi J.J., Balko J.M. (2022). Immune-checkpoint inhibitors: Long-term implications of toxicity. Nat. Rev. Clin. Oncol..

[19] Wright J.J., Salem J.E., Johnson D.B., Lebrun-Vignes B., Stamatouli A., Thomas J.W., Herold K.C., Moslehi J., Powers A.C. (2018). Increased reporting of immune checkpoint inhibitor-associated diabetes. Diabetes Care.

[20] Lee D., Lee H.J., Farmer J.R., Reynolds K.L. (2021). Mechanisms Driving Immune-Related Adverse Events in Cancer Patients Treated with Immune Checkpoint Inhibitors. Curr. Cardiol. Rep..

[21] Durgeau A., Virk Y., Corgnac S., Mami-Chouaib F. (2018). Recent Advances in Targeting CD8 T-Cell Immunity for More Effective Cancer Immunotherapy. Front. Immunol..

[22] Willsmore Z.N., Harris R.J., Crescioli S., Hussein K., Kakkassery H., Thapa D., Cheung A., Chauhan J., Bax H.J., Chenoweth A. (2020). B Cells in Patients with Melanoma: Implications for Treatment with Checkpoint Inhibitor Antibodies. Front. Immunol..

[23] Pavan A., Calvetti L., Dal Maso A., Attili I., Del Bianco P., Pasello G., Guarneri V., Aprile G., Conte P., Bonanno L. (2019). Peripheral Blood Markers Identify Risk of Immune-Related Toxicity in Advanced Non-Small Cell Lung Cancer Treated with Immune-Checkpoint Inhibitors. Oncologist.

[24] Lim S.Y., Lee J.H., Gide T.N., Menzies A.M., Guminski A., Carlino M.S., Breen E.J., Yang J.Y.H., Ghazanfar S., Kefford R.F. (2019). Circulating Cytokines Predict Immune-Related Toxicity in Melanoma Patients Receiving Anti-PD-1-based Immunotherapy. Clin. Cancer Res..

[25] Jia X.H., Geng L.Y., Jiang P.P., Xu H., Nan K.J., Yao Y., Jiang L.L., Sun H., Qin T.J., Guo H. (2020). The biomarkers related to immune related adverse events caused by immune checkpoint inhibitors. J. Exp. Clin. Cancer Res..

[26] Mor A., Strazza M. (2022). Bridging the Gap: Connecting the Mechanisms of Immune-Related Adverse Events and Autoimmunity Through PD-1. Front. Cell Dev. Biol..

[27] Carr A.L.J., Evans-Molina C., Oram R.A. (2022). Precision medicine in type 1 diabetes. Diabetologia.

[28] Mota Reyes C., Demir E., Çifcibaşı K., Istvanffy R., Friess H., Demir I.E. (2022). Regulatory T Cells in Pancreatic Cancer: Of Mice and Men. Cancers.

[29] Shevyrev D., Tereshchenko V. (2020). Treg Heterogeneity, Function, and Homeostasis. Front. Immunol..

[30] Viisanen T., Gazali A.M., Ihantola E.L., Ekman I., Näntö-Salonen K., Veijola R., Toppari J., Knip M., Ilonen J., Kinnunen T. (2019). FOXP3+ Regulatory T Cell Compartment Is Altered in Children with Newly Diagnosed Type 1 Diabetes but Not in Autoantibody-Positive at-Risk Children. Front. Immunol..

[31] Anderson A.M., Landry L.G., Alkanani A.A., Pyle L., Powers A.C., Atkinson M.A., Mathews C.E., Roep B.O., Michels A.W., Nakayama M. (2021). Human islet T cells are highly reactive to preproinsulin in type 1 diabetes. Proc. Natl. Acad. Sci. USA.

[32] Insel R.A., Dunne J.L., Atkinson M.A., Chiang J.L., Dabelea D., Gottlieb P.A., Greenbaum C.J., Herold K.C., Krischer J.P., Lernmark Å. (2015). Staging presymptomatic type 1 diabetes: A scientific statement of JDRF, the Endocrine Society, and the American Diabetes Association. Diabetes Care.

[33] Ogando J., Saez M.E., Santos J., Nuevo-Tapioles C., Gut M., Esteve-Codina A., Heath S., Gonzalez-Perez A., Cuezva J.M., Lacalle R.A. (2019). PD-1 signaling affects cristae morphology and leads to mitochondrial dysfunction in human CD8(+) T lymphocytes. J. Immunother. Cancer.

[34] Pichler A.C., Cannons J.L., Schwartzberg P.L. (2022). The Road Less Taken: Less Appreciated Pathways for Manipulating CD8^+^ T Cell Exhaustion. Front. Immunol..

[35] He X., Xu C. (2020). PD-1: A Driver or Passenger of T Cell Exhaustion?. Mol. Cell.

[36] Gao Z., Feng Y., Xu J., Liang J. (2022). T-cell exhaustion in immune-mediated inflammatory diseases: New implications for immunotherapy. Front. Immunol..

[37] Linsley P.S., Long S.A. (2019). Enforcing the checkpoints: Harnessing T-cell exhaustion for therapy of T1D. Curr. Opin. Endocrinol. Diabetes Obes..

[38] Colli M.L., Hill J.L.E., Marroquí L., Chaffey J., Dos Santos R.S., Leete P., Coomand de Brachѐne A., Paula F.M.M., Op de Beeck A., Castela A. (2018). PDL1 is expressed in the islets of people with type 1 diabetes and is up-regulated by interferons-α and-γ via IRF1 induction. Ebiomedicine.

[39] Osum K.C., Burrack A.L., Martinov T., Sahli N.L., Mitchell J.S., Tucker C.G., Pauken K.E., Papas K., Appakalai B., Spanier J.A. (2018). Interferon-gamma drives programmed death-ligand 1 expression on islet β cells to limit T cell function during autoimmune diabetes. Sci. Rep..

[40] Kwong C.J., Selck C., Tahija K., McAnaney L.J., Le D.V., Kay T.W., Thomas H.E., Krishnamurthy B. (2021). Harnessing CD8^+^ T-cell exhaustion to treat type 1 diabetes. Immunol. Cell Biol..

[41] Wong F.S., Wen L. (2020). A predictive CD8^+^ T cell phenotype for T1DM progression. Nat. Rev. Endocrinol..

[42] Wiedeman A.E., Muir V.S., Rosasco M.G., DeBerg H.A., Presnell S., Haas B., Dufort M.J., Speake C., Greenbaum C.J., Serti E. (2020). Autoreactive Cd8^+^ T cell exhaustion distinguishes subjects with slow type 1 diabetes progression. J. Clin. Investig..

[43] Shan Y., Kong Y., Zhou Y., Guo J., Shi Q., Li S., Guo H., Huang Y., Ding S., Liu C. (2021). Decreased expression of programmed death-1 on CD8^+^ effector memory T lymphocytes correlates with the pathogenesis of type 1 diabetes. Acta Diabetol..

[44] Pellegrino M., Crinò A., Rosado M.M., Fierabracci A. (2019). Identification and functional characterization of CD8^+^ T regulatory cells in type 1 diabetes patients. PLoS ONE.

[45] Zagouras A., Patil P.D., Yogi-Morren D., Pennell N.A. (2020). Cases from the Immune-Related Adverse Event Tumor Board: Diagnosis and Management of Immune Checkpoint Blockade-Induced Diabetes. Oncologist.

[46] Yoneda S., Imagawa A., Hosokawa Y., Baden M.Y., Kimura T., Uno S., Fukui K., Goto K., Uemura M., Eguchi H. (2019). T-Lymphocyte Infiltration to Islets in the Pancreas of a Patient Who Developed Type 1 Diabetes After Administration of Immune Checkpoint Inhibitors. Diabetes Care.

[47] Mourad D., Azar N.S., Eid A.A., Azar S.T. (2021). Immune Checkpoint Inhibitor-Induced Diabetes Mellitus: Potential Role of T Cells in the Underlying Mechanism. Int. J. Mol. Sci..

[48] Mazzucato M., Garelli S., Betterle C., Presotto F. (2020). Checkpoint inhibitor develops histological autoimmune pancreatitis like type 1 diabetes. A case report. MOJ Clin. Med. Case Rep..

[49] Redondo M.J., Steck A.K., Pugliese A. (2018). Genetics of type 1 diabetes. Pediatr. Diabetes.

[50] Zhao L.P., Papadopoulos G.K., Moustakas A.K., Bondinas G.P., Carlsson A., Larsson H.E., Ludvigsson J., Marcus C., Persson M., Samuelsson U. (2021). Nine residues in HLA-DQ molecules determine with susceptibility and resistance to type 1 diabetes among young children in Sweden. Sci. Rep..

[51] Sticht J., Álvaro-Benito M., Konigorski S. (2021). Type 1 Diabetes and the HLA Region: Genetic Association Besides Classical HLA Class II Genes. Front. Genet..

[52] Stamatouli A.M., Quandt Z., Perdigoto A.L., Clark P.L., Kluger H., Weiss S.A., Gettinger S., Sznol M., Young A., Rushakoff R. (2018). Collateral damage: Insulin-dependent diabetes induced with checkpoint inhibitors. Diabetes.

[53] Tsang V.H.M., McGrath R.T., Clifton-Bligh R.J., Scolyer R.A., Jakrot V., Guminski A.D., Long G.V., Menzies A.M. (2019). Checkpoint Inhibitor-Associated Autoimmune Diabetes Is Distinct from Type 1 Diabetes. J. Clin. Endocrinol. Metab..

[54] Yun K., Daniels G., Gold K., McCowen K., Patel S.P. (2020). Rapid Onset Type 1 Diabetes with Anti-PD-1 Directed Therapy. Oncotarget.

[55] Akturk H.K., Kahramangil D., Sarwal A., Hoffecker L., Murad M.H., Michels A.W. (2019). Immune checkpoint inhibitor-induced Type 1 diabetes: A systematic review and meta-analysis. Diabet. Med..

[56] Lo Preiato V., Salvagni S., Ricci C., Ardizzoni A., Pagotto U., Pelusi C. (2021). Diabetes Mellitus Induced by Immune Checkpoint Inhibitors: Type 1 Diabetes Variant or New Clinical Entity?. Rev. Lit. Rev. Endocr. Metab. Disord..

[57] Wen S., Jiang W., Zhou L. (2021). Islet Autoantibodies in the Patients with Sjogren’s Syndrome and Thyroid Disease and Risk of Progression to Latent Autoimmune Diabetes in Adults: A Case Series. Diabetes Metab. Syndr. Obes..

[58] Mistry S., Gouripeddi R., Raman V., Facelli J.C. (2022). Stratifying risk for onset of type 1 diabetes using islet autoantibody trajectory clustering. Diabetologia.

[59] Mallone R., Eizirik D.L. (2020). Presumption of innocence for beta cells: Why are they vulnerable autoimmune targets in type 1 diabetes?. Diabetologia.

[60] Hamadi G.M. (2022). Immunological markers in type 1 diabetes mellitus in Thi-Qar province, southern Iraq. J. Med. Life.

[61] De Filette J.M.K., Pen J.J., Decoster L., Vissers T., Bravenboer B., van der Auwera B.J., Gorus F.K., Roep B.O., Aspeslagh S., Neyns B. (2019). Immune Checkpoint Inhibitors and Type 1 Diabetes Mellitus: A Case Report and Systematic Review. Eur. J. Endocrinol..

[62] Kotwal A., Haddox C., Block M., Kudva Y.C. (2019). Immune Checkpoint Inhibitors: An Emerging Cause of Insulin-Dependent Diabetes. BMJ Open Diabetes Res. Care.

[63] Tan M.H., Iyengar R., Mizokami-Stout K., Yentz S., MacEachern M.P., Shen L.Y., Redman B., Gianchandani R. (2019). Spectrum of immune checkpoint inhibitors-induced endocrinopathies in cancer patients: A scoping review of case reports. Clin. Diabetes Endocrinol..

[64] Huang X., Yang M., Wang L., Li L., Zhong X. (2021). Sintilimab induced diabetic ketoacidosis in a patient with small cell lung cancer: A case report and literature review. Medicine.

[65] Martinov T., Swanson L.A., Breed E.R., Tucker C.G., Dwyer A.J., Johnson J.K., Mitchell J.S., Sahli N.L., Wilson J.C., Singh L.M. (2019). Programmed Death-1 Restrains the Germinal Center in Type 1 Diabetes. J. Immunol..

[66] Hao H., Nakayamada S., Tanaka Y. (2021). Differentiation, functions, and roles of T follicular regulatory cells in autoimmune diseases. Inflamm. Regen..

[67] Xu X., Shen M., Zhao R., Cai Y., Jiang H., Shen Z., Gao R., Xu K., Chen H., Yang T. (2019). Follicular regulatory T cells are associated with β-cell autoimmunity and the development of type 1 diabetes. J. Clin. Endocrinol. Metab..

[68] Lu Y., Craft J. (2021). T Follicular Regulatory Cells: Choreographers of Productive Germinal Center Responses. Front. Immunol..

[69] Matijašić M., Meštrović T., Paljetak H.Č., Perić M., Barešić A., Verbanac D. (2020). Gut Microbiota beyond Bacteria-Mycobiome, Virome, Archaeome, and Eukaryotic Parasites in IBD. Int. J. Mol. Sci..

[70] Zhang T., Gao G., Sakandar H.A., Kwok L.Y., Sun Z. (2022). Gut Dysbiosis in Pancreatic Diseases: A Causative Factor and a Novel Therapeutic Target. Front. Nutr..

[71] Shilo S., Godneva A., Rachmiel M., Korem T., Bussi Y., Kolobkov D., Karady T., Bar N., Wolf B.C., Glantz-Gashai Y. (2022). The Gut Microbiome of Adults with Type 1 Diabetes and Its Association with the Host Glycemic Control. Diabetes Care.

[72] van Heck J.I.P., Gacesa R., Stienstra R., Fu J., Zhernakova A., Harmsen H.J.M., Weersma R.K., Joosten L.A.B., Tack C.J. (2022). The Gut Microbiome Composition Is Altered in Long-standing Type 1 Diabetes and Associates with Glycemic Control and Disease-Related Complications. Diabetes Care.

[73] Gopalakrishnan V., Spencer C.N., Nezi L., Reuben A., Andrews M.C., Karpinets T.V., Prieto P.A., Vicente D., Hoffman K., Wei S.C. (2018). Gut microbiome modulates response to anti-PD-1 immunotherapy in melanoma patients. Science.

[74] Dai Z., Zhang J., Wu Q., Fang H., Shi C., Li Z., Lin C., Tang D., Wang D. (2020). Intestinal microbiota: A new force in cancer immunotherapy. Cell Commun. Signal.

[75] Oey O., Liu Y.Y., Sunjaya A.F., Simadibrata D.M., Khattak M.A., Gray E. (2022). Gut microbiota diversity and composition in predicting immunotherapy response and immunotherapy-related colitis in melanoma patients: A systematic review. World J. Clin. Oncol..

[76] Oh B., Boyle F., Pavlakis N., Clarke S., Eade T., Hruby G., Lamoury G., Carroll S., Morgia M., Kneebone A. (2021). The Gut Microbiome and Cancer Immunotherapy: Can We Use the Gut Microbiome as a Predictive Biomarker for Clinical Response in Cancer Immunotherapy?. Cancers.

[77] Martínez M.S., Manzano A., Olivar L.C., Nava M., Salazar J., D’Marco L., Ortiz R., Chacín M., Guerrero-Wyss M., Cabrera de Bravo M. (2021). The Role of the α Cell in the Pathogenesis of Diabetes: A World beyond the Mirror. Int. J. Mol. Sci..

[78] Ross J.J., Wasserfall C.H., Bacher R., Perry D.J., McGrail K., Posgai A.L., Dong X., Muir A., Li X., Campbell-Thompson M. (2021). Exocrine Pancreatic Enzymes Are a Serological Biomarker for Type 1 Diabetes Staging and Pancreas Size. Diabetes.

[79] Ko J., Cho J., Petrov M.S. (2020). Low serum amylase, lipase, and trypsin as biomarkers of metabolic disorders: A systematic review and meta-analysis. Diabetes Res. Clin. Pract..

[80] Marchand L., Thivolet A., Dalle S., Chikh K., Reffet S., Vouillarmet J., Fabien N., Cugnet-Anceau C., Thivolet C. (2019). Diabetes mellitus induced by PD-1 and PD-L1 inhibitors: Description of pancreatic endocrine and exocrine phenotype. Acta Diabetol..

[81] Marchand L., Thivolet A., Saintigny P., Fabien N., Vouillarmet J., Thivolet C. (2018). Anti-Programmed Death 1 (PD-1) Antibodies and the Pancreas: A Diabetic Storm Ahead?. Diabetes Care.

[82] Nakano R., Shiomi H., Fujiwara A., Yoshihara K., Yoshioka R., Kawata S., Ota S., Yuri Y., Takashima T., Aizawa N. (2022). Clinical Characteristics of ICI-Related Pancreatitis and Cholangitis Including Radiographic and Endoscopic Findings. Healthcare.

[83] George J., Bajaj D., Sankaramangalam K., Yoo J.W., Joshi N.S., Gettinger S., Price C., Farrell J.J. (2019). Incidence of pancreatitis with the use of immune checkpoint inhibitors (ICI) in advanced cancers: A systematic review and meta-analysis. Pancreatology.

[84] Su Q., Zhang X.C., Zhang C.G., Hou Y.L., Yao Y.X., Cao B.W. (2018). Risk of Immune-Related Pancreatitis in Patients with Solid Tumors Treated with Immune Checkpoint Inhibitors: Systematic Assessment with Meta-Analysis. J. Immunol. Res..

[85] Zhang T., Wang Y., Shi C., Liu X., Lv S., Wang X., Li W. (2022). Pancreatic injury following immune checkpoint inhibitors: A systematic review and meta-analysis. Front. Pharmacol..

[86] Corvillo F., Aparicio V., López-Lera A., Garrido S., Araújo-Vilar D., de Miguel M.P., López-Trascasa M. (2018). Autoantibodies Against Perilipin 1 as a Cause of Acquired Generalized Lipodystrophy. Front. Immunol..

[87] Koethe J.R., Lagathu C., Lake J.E., Domingo P., Calmy A., Falutz J., Brown T.T., Capeau J. (2020). HIV and antiretroviral therapy-related fat alterations. Nat. Rev. Dis. Primers.

[88] Falcao C.K., Cabral M.C.S., Mota J.M., Arbache S.T., Costa-Riquetto A.D., Muniz D.Q.B., Cury-Martins J., Almeida M.Q., Kaczemorska P.C., Nery M. (2019). Acquired Lipodystrophy Associated with Nivolumab in a Patient With Advanced Renal Cell Carcinoma. J. Clin. Endocrinol. Metab..

[89] Haddad N., Vidal-Trecan T., Baroudjian B., Zagdanski A.M., Arangalage D., Battistella M., Gautier J.F., Lebbe C., Delyon J., PATIO Group (2020). Acquired generalized lipodystrophy under immune checkpoint inhibition. Br. J. Dermatol..

[90] Bedrose S., Turin C.G., Lavis V.R., Kim S.T., Thosani S.N. (2020). A Case of Acquired Generalized Lipodystrophy Associated with Pembrolizumab in a Patient with Metastatic Malignant Melanoma. AACE Clin. Case Rep..

[91] Jehl A., Cugnet-Anceau C., Vigouroux C., Legeay A.L., Dalle S., Harou O., Marchand L., Lascols O., Caussy C., Thivolet C. (2019). Acquired Generalized Lipodystrophy: A New Cause of Anti-PD-1 Immune-Related Diabetes. Diabetes Care.

[92] Mandel-Brehm C., Vazquez S.E., Liverman C., Cheng M., Quandt Z., Kung A.F., Parent A., Miao B., Disse E., Cugnet-Anceau C. (2023). Autoantibodies to Perilipin-1 Define a Subset of Acquired Generalized Lipodystrophy. Diabetes.

[93] Kalra S. (2018). Post-immunotherapy new onset diabetes (PINOD)-under-recognized etiology, unexplored presentation. Ann. Transl. Med..

[94] Barroso-Sousa R., Barry W.T., Garrido-Castro A.C., Hodi F.S., Min L., Krop I.E., Tolaney S.M. (2018). Incidence of Endocrine Dysfunction Following the Use of Different Immune Checkpoint Inhibitor Regimens a Systematic Review and Meta-Analysis. JAMA Oncol..

[95] Bajwa R., Cheema A., Khan T., Amirpour A., Paul A., Chaughtai S., Patel S., Patel T., Bramson J., Gupta V. (2019). Adverse Effects of Immune Checkpoint Inhibitors (Programmed Death-1 Inhibitors and Cytotoxic T-Lymphocyte-Associated Protein-4 Inhibitors): Results of a Retrospective Study. J. Clin. Med. Res..

[96] Xu H., Tan P., Zheng X., Huang Y., Lin T., Wei Q., Ai J., Yang L. (2019). Immune-related adverse events following administration of anti-cytotoxic T-lymphocyte-associated protein-4 drugs: A comprehensive systematic review and meta-analysis. Drug Des. Devel. Ther..

[97] Lu J., Yang J., Liang Y., Meng H., Zhao J., Zhang X. (2019). Incidence of Immune Checkpoint Inhibitor-Associated Diabetes: A Meta-Analysis of Randomized Controlled Studies. Front. Pharmacol..

[98] Ji H.H., Tang X.W., Dong Z., Song L., Jia Y.T. (2019). Adverse Event Profiles of Anti-CTLA-4 and Anti-PD-1 Monoclonal Antibodies Alone or in Combination: Analysis of Spontaneous Reports Submitted to FAERS. Clin. Drug Investig..

[99] Zhai Y., Ye X., Hu F., Xu J., Guo X., Zhuang Y., He J. (2019). Endocrine toxicity of immune checkpoint inhibitors: A real-world study leveraging US Food and Drug Administration adverse events reporting system. J. Immunother. Cancer.

[100] Chen X., Affinati A.H., Lee Y., Turcu A.F., Henry N.L., Schiopu E., Qin A., Othus M., Clauw D., Ramnath N. (2022). Immune Checkpoint Inhibitors and Risk of Type 1 Diabetes. Diabetes Care.

[101] Perdigoto A.L., Quandt Z., Anderson M., Herold K.C. (2019). Checkpoint inhibitor-induced insulin-dependent diabetes: An emerging syndrome. Lancet Diabetes Endocrinol..

[102] Rodríguez de Vera-Gómez P., Piñar-Gutiérrez A., Guerrero-Vázquez R., Bellido V., Morales-Portillo C., Sancho-Márquez M.P., Espejo-García P., Gros-Herguido N., López-Gallardo G., Martínez-Brocca M.A. (2022). Flash Glucose Monitoring and Diabetes Mellitus Induced by Immune Checkpoint Inhibitors: An Approach to Clinical Practice. J. Diabetes Res..

[103] Byun D.J., Braunstein R., Flynn J., Zheng J., Lefkowitz R.A., Kanbour S., Girotra M. (2020). Immune Checkpoint Inhibitor–Associated Diabetes: A Single-Institution Experience. Diabetes Care.

[104] Liu J., Shi Y., Liu X., Zhang D., Zhang H., Chen M., Xu Y., Zhao J., Zhong W., Wang M. (2022). Clinical characteristics and outcomes of immune checkpoint inhibitor-induced diabetes mellitus. Transl. Oncol..

[105] Kyriacou A., Melson E., Chen W., Kempegowda P. (2020). Is immune checkpoint inhibitor-associated diabetes the same as fulminant type 1 diabetes mellitus?. Clin. Med..

[106] Brahmer J.R., Lacchetti C., Schneider B.J., Atkins M.B., Brassil K.J., Caterino J.M., Chau I., Ernstoff M.S., Gardner J.M., Ginex P. (2018). National Comprehensive Cancer Network (2018). Management of Immune-Related Adverse Events in Patients Treated with Immune Checkpoint Inhibitor Therapy: American Society of Clinical Oncology Clinical Practice Guideline. J. Clin. Oncol..

[107] Smati S., Buffier P., Bouillet B., Archambeaud F., Vergès B., Cariou B. (2018). Expert opinion on immunotherapy induced diabetes. Ann. Endocrinol..

[108] Castinetti F., Albarel F., Archambeaud F., Bertherat J., Bouillet B., Buffier P., Briet C., Cariou B., Caron P., Chabre O. (2019). French Endocrine Society Guidance on endocrine side effects of immunotherapy. Endocr. Relat. Cancer.

[109] Holt R.I.G., DeVries J.H., Hess-Fischl A., Hirsch I.B., Kirkman M.S., Klupa T., Ludwig B., Nørgaard K., Pettus J., Renard E. (2021). The Management of Type 1 Diabetes in Adults. A Consensus Report by the American Diabetes Association (ADA) and the European Association for the Study of Diabetes (EASD). Diabetes Care.

[110] Schneider B.J., Naidoo J., Santomasso B.D., Lacchetti C., Adkins S., Anadkat M., Atkins M.B., Brassil K.J., Caterino J.M., Chau I. (2021). Management of Immune-Related Adverse Events in Patients Treated with Immune Checkpoint Inhibitor Therapy: ASCO Guideline Update. J. Clin. Oncol..

[111] Dhatariya K.K., Joint British Diabetes Societies for Inpatient Care (2022). The management of diabetic ketoacidosis in adults—An updated guideline from the Joint British Diabetes Society for Inpatient Care. Diabet. Med..

[112] Eledrisi M.S., Elzouki A.N. (2020). Management of Diabetic Ketoacidosis in Adults: A Narrative Review. Saudi J. Med. Med. Sci..

[113] Imagawa A., Hanafusa T. (2011). Fulminant type 1 diabetes—An important subtype in East Asia. Diabetes Metab. Res. Rev..

[114] Roy A., Sahoo J., Kamalanathan S., Naik D., Mohan P., Kalayarasan R. (2021). Diabetes and pancreatic cancer: Exploring the two-way traffic. World J. Gastroenterol..

[115] Duan X., Wang W., Pan Q., Guo L. (2021). Type 2 Diabetes Mellitus Intersects with Pancreatic Cancer Diagnosis and Development. Front. Oncol..

[116] Yim C., Mansell K., Hussein N., Arnason T. (2021). Current cancer therapies and their influence on glucose control. World J. Diabetes..

[117] Joharatnam-Hogan N., Carter T.J., Reynolds N., Ho J.H., Adam S., Board R., Feingold K.R., Anawalt B., Blackman M.R., Boyce A., Chrousos G., Corpas E., de Herder W.W., Dhatariya K., Dungan K., Hofland J. (2000). Diabetes Mellitus in People with Cancer. Endotext.

[118] Pinheiro L.C., Kaur H., Nilo D., Safford M.M., DeRosa A.P., Kern L.M. (2019). Determining the Impact of a Cancer Diagnosis on Diabetes Management: A Systematic Literature Review. Am. J. Clin. Oncol..

[119] American Diabetes Association (2020). Standards of Medical Care in Diabetes—2020; Abridged for Primary Care Providers. Clin. Diabetes.

[120] Brahmer J.R., Abu-Sbeih H., Ascierto P.A., Brufsky J., Cappelli L.C., Cortazar F.B., Gerber D.E., Hamad L., Hansen E., Johnson D.B. (2021). Society for Immunotherapy of Cancer (SITC) clinical practice guideline on immune checkpoint inhibitor-related adverse events. J. Immunother. Cancer.

[121] Okubo M., Hataya Y., Fujimoto K., Iwakura T., Matsuoka N. (2023). Recovery from insulin dependence in immune checkpoint inhibitor-associated diabetes mellitus: A case report. J. Diabetes Investig..

[122] Chennamadhavuni A., Abushahin L., Jin N., Presley C.J., Manne A. (2022). Risk Factors and Biomarkers for Immune-Related Adverse Events: A Practical Guide to Identifying High-Risk Patients and Rechallenging Immune Checkpoint Inhibitors. Front. Immunol..

[123] Allouchery M., Lombard T., Martin M., Rouby F., Sassier M., Bertin C., Atzenhoffer M., Miremont-Salame G., Perault-Pochat M.C., Puyade M. (2020). Safety of immune checkpoint inhibitor rechallenge after discontinuation for grade ≥ 2 immune-related adverse events in patients with cancer. J. Immunother. Cancer.

[124] Simonaggio A., Michot J.M., Voisin A.L., Le Pavec J., Collins M., Lallart A., Cengizalp G., Vozy A., Laparra A., Varga A. (2019). Evaluation of Readministration of Immune Checkpoint Inhibitors After Immune-Related Adverse Events in Patients with Cancer. JAMA Oncol..

[125] Haratani K., Hayashi H., Nakagawa K. (2020). Association of immune-related adverse events with immune checkpoint inhibitor efficacy: Real or imaginary?. BMC Med..

[126] Akamatsu H., Murakami E., Oyanagi J., Shibaki R., Kaki T., Takase E., Tanaka M., Harutani Y., Yamagata N., Okuda Y. (2020). Immune-Related Adverse Events by Immune Checkpoint Inhibitors Significantly Predict Durable Efficacy Even in Responders with Advanced Non-Small Cell Lung Cancer. Oncologist.

[127] Zhou X., Yao Z., Yang H., Liang N., Zhang X., Zhang F. (2020). Are immune-related adverse events associated with the efficacy of immune checkpoint inhibitors in patients with cancer? A systematic review and meta analysis. BMC Med..

[128] Rogado J., Sánchez-Torres J.M., Romero-Laorden N., Ballesteros A.I., Pacheco-Barcia V., Ramos-Leví A., Arranz R., Lorenzo A., Gullón P., Donnay O. (2019). Immune-related adverse events predict the therapeutic efficacy of anti-PD-1 antibodies in cancer patients. Eur. J. Cancer.

[129] Wang D., Chen C., Gu Y., Lu W., Zhan P., Liu H., Lv T., Song Y., Zhang F. (2021). Immune-Related Adverse Events Predict the Efficacy of Immune Checkpoint Inhibitors in Lung Cancer Patients: A Meta-Analysis. Front. Oncol..

[130] Gunjur A., Klein O., Kee D., Cebon J. (2019). Anti-programmed cell death protein 1 (anti-PD1) immunotherapy induced autoimmune polyendocrine syndrome type II (APS-2): A case report and review of the literature. J. Immunother. Cancer.

[131] Ebrahim E., Teklu T., Tajebe F., Wondmagegn T., Akelew Y., Fiseha M. (2022). Association of Cytotoxic T-Lymphocyte Antigen-4 Gene Polymorphism with Type 1 Diabetes Mellitus: In silico Analysis of Biological Features of CTLA-4 Protein on Ethiopian Population. Diabetes Metab. Syndr. Obes..

[132] Chen Y., Chen S., Gu Y., Feng Y., Shi Y., Fu Q., Wang Z., Cai Y., Dai H., Zheng S. (2018). CTLA-4 +49 G/A, a functional T1D risk SNP, affects CTLA-4 level in Treg subsets and IA-2A positivity, but not beta-cell function. Sci. Rep..

[133] Ren D., He L., Pang X. (2022). Association of CLTA-4 Gene Polymorphisms with Diabetes Mellitus: A Study Based on the Han Population of Northern China. Diabetes Metab. Syndr Obes..

[134] Galicia-Garcia U., Benito-Vicente A., Jebari S., Larrea-Sebal A., Siddiqi H., Uribe K.B., Ostolaza H., Martín C. (2020). Pathophysiology of Type 2 Diabetes Mellitus. Int. J. Mol. Sci..

[135] Goodarzi M.O., Petrov M.S., Andersen D.K., Hart P.A. (2021). Diabetes in chronic pancreatitis: Risk factors and natural history. Curr. Opin. Gastroenterol..

[136] Xu Y., Fu Y., Zhu B., Wang J., Zhang B. (2020). Predictive Biomarkers of Immune Checkpoint Inhibitors-Related Toxicities. Front. Immunol..

[137] Curran C.S., Sommers C.L., Young H.A., Bourcier K., Mancini M., Sharon E. (2019). Report on the 2018 Cancer, Autoimmunity, and Immunology Conference. J. Immunol..

[138] Nixon A.B., Schalper K.A., Jacobs I., Potluri S., Wang I.M., Fleener C. (2019). Peripheral immune-based biomarkers in cancer immunotherapy: Can we realize their predictive potential?. J. Immunother. Cancer.

[139] Januszewski A.S., Cho Y.H., Joglekar M.V., Farr R.J., Scott E.S., Wong W.K.M., Carroll L.M., Loh Y.W., Benitez-Aguirre P.Z., Keech A.C. (2021). Insulin micro-secretion in Type 1 diabetes and related microRNA profiles. Sci. Rep..

[140] Tanvir Ahmed K., Cheng S., Li Q., Yong J., Zhang W. (2023). Incomplete time-series gene expression in integrative study for islet autoimmunity prediction. Brief. Bioinform..

[141] Gowen M.F., Giles K.M., Simpson D., Tchack J., Zhou H., Moran U., Dawood Z., Pavlick A.C., Hu S., Wilson M.A. (2018). Baseline antibody profiles predict toxicity in melanoma patients treated with immune checkpoint inhibitors. J. Transl. Med..

[142] Hassel J.C., Luke J.J. (2022). Autoantibodies as Predictors for Clinical Outcome and Toxicity for Immunotherapy. Clin. Cancer Res..

[143] Johannet P., Liu W., Fenyo D., Wind-Rotolo M., Krogsgaard M., Mehnert J.M., Weber J.S., Zhong J., Osman I. (2022). Baseline Serum Autoantibody Signatures Predict Recurrence and Toxicity in Melanoma Patients Receiving Adjuvant Immune Checkpoint Blockade. Clin. Cancer Res..

[144] Redondo M.J., Geyer S., Steck A.K., Sharp S., Wentworth J.M., Weedon M.N., Antinozzi P., Sosenko J., Atkinson M., Pugliese A. (2018). Type 1 Diabetes TrialNet Study Group. A Type 1 Diabetes Genetic Risk Score Predicts Progression of Islet Autoimmunity and Development of Type 1 Diabetes in Individuals at Risk. Diabetes Care.

[145] Khan Z., Hammer C., Guardino E., Chandler G.S., Albert M.L. (2019). Mechanisms of immune-related adverse events associated with immune checkpoint blockade: Using germline genetics to develop a personalized approach. Genome Med..

[146] Cai Q., Huo G.W., Zhu F.Y., Yue P., Yuan D.Q., Chen P. (2022). Safety and efficacy of immune checkpoint inhibitors in advanced cancer patients with autoimmune disease: A meta-analysis. Hum. Vaccin. Immunother..

[147] Heinzerling L., Ascierto P.A., Dummer R., Gogas H., Grob J.J., Lebbe C., Long G.V., McArthur G., Moslehi J.J., Neilan T.G. (2019). Adverse events 2.0-Let us get SERIOs: New reporting for adverse event outcomes needed in the era of immuno-oncology. Eur. J. Cancer.

[148] Nuzzo P.V., Pond G.R., Abou Alaiwi S., Nassar A.H., Flippot R., Curran C., Kilbridge K.L., Wei X.X., McGregor B.A., Choueiri T. (2020). Conditional immune toxicity rate in patients with metastatic renal and urothelial cancer treated with immune checkpoint inhibitors. J. Immunother. Cancer.

[149] Das S., Johnson D.B. (2019). Immune-related adverse events, and anti-tumor efficacy of immune checkpoint inhibitors. J. Immunother. Cancer.

[150] Martins F., Sykiotis G.P., Maillard M., Fraga M., Ribi C., Kuntzer T., Michielin O., Peters S., Coukos G., Spertini F. (2019). New therapeutic perspectives to manage refractory immune checkpoint-related toxicities. Lancet Oncol..

[151] Arutyunyan I.V., Fatkhudinov T.K., Makarov A.V., Elchaninov A.V., Sukhikh G.T. (2020). Regenerative medicine of pancreatic islets. World J. Gastroenterol..

[152] Porter J.M., Guerassimoff L., Castiello F.R., Charette A., Tabrizian M. (2022). INGAP-Peptide Variants as a Novel Therapy for Type 1 Diabetes: Effect on Human Islet Insulin Secretion and Gene Expression. Pharmaceutics.

[153] Zhan Z.T., Liu L., Cheng M.Z., Gao Y., Zhou W.J. (2022). The Effects of 6 Common Antidiabetic Drugs on Anti-PD1 Immune Checkpoint Inhibitor in Tumor Treatment. J. Immunol. Res..

[154] Hu W., Wang G., Wang Y., Riese M.J., You M. (2020). Uncoupling Therapeutic Efficacy from Immune-Related Adverse Events in Immune Checkpoint Blockade. iScience.

[155] Jin S., Sun Y., Liang X., Gu X., Ning J., Xu Y., Chen S., Pan L. (2022). Emerging new therapeutic antibody derivatives for cancer treatment. Signal Transduct. Target Ther..

[156] Thoreau F., Chudasama V. (2021). Enabling the next steps in cancer immunotherapy: From antibody-based bispecifics to multispecifics, with an evolving role for bioconjugation chemistry. RSC Chem. Biol..

[157] Jacobi O., Landman Y., Reinhorn D., Icht O., Sternschuss M., Rotem O., Finkel I., Allen A.M., Dudnik E., Goldstein D.A. (2021). The Relationship of Diabetes Mellitus to Efficacy of Immune Checkpoint Inhibitors in Patients with Advanced Non-Small Cell Lung Cancer. Oncology.

[158] Shahid R.K., Ahmed S., Le D., Yadav S. (2021). Diabetes and Cancer: Risk, Challenges, Management and Outcomes. Cancers.

